# Mesoporous Silica Materials as an Emerging Tool for Cancer Immunotherapy

**DOI:** 10.1002/advs.202200756

**Published:** 2022-07-22

**Authors:** Blanca Escriche‐Navarro, Andrea Escudero, Elena Lucena‐Sánchez, Félix Sancenón, Alba García‐Fernández, Ramón Martínez‐Máñez

**Affiliations:** ^1^ Interuniversity Research Institute for Molecular Recognition and Technological Development (IDM) Polytechnic University of Valencia‐University of Valencia Camino de Vera s/n Valencia 46022 Spain; ^2^ Universitat Politècnica de València Joint Unit UPV‐CIPF of Developmental Biology and Disease Models and Nanomedicine, Polytechnic University of Valencia (UPV)‐Príncipe Felipe Research Center Foundation (CIPF) C/ Eduardo Primo Yúfera 3. Valencia 46012 Spain; ^3^ Joint Unit of Nanomedicine and Sensors, Polytechnic University of Valencia, IIS La Fe Av. Fernando Abril Martorell, 106 Valencia 46026 Spain; ^4^ Biomedical Research Networking Center in Bioengineering, Biomaterials and Nanomedicine (CIBER‐BBN) Av. Monforte de Lemos, 3–5. Pabellón 11., Planta 0 Madrid 28029 Spain

**Keywords:** cancer, checkpoint inhibitors, immunotherapy, mesoporous silica nanoparticles, photodynamic therapies, vaccines

## Abstract

Cancer immunotherapy has emerged in the past decade as a promising strategy for treating many forms of cancer by stimulating the patient's immune system. Although immunotherapy has achieved some promising results in clinics, more efforts are required to improve the limitations of current treatments related to lack of effective and targeted cancer antigens delivery to immune cells, dose‐limiting toxicity, and immune‐mediated adverse effects, among others. In recent years, the use of nanomaterials has proven promising to enhance cancer immunotherapy efficacy and reduce side effects. Among nanomaterials, attention has been recently paid to mesoporous silica nanoparticles (MSNs) as a potential multiplatform for enhancing cancer immunotherapy by considering their unique properties, such as high porosity, and good biocompatibility, facile surface modification, and self‐adjuvanticity. This review explores the role of MSN and other nano/micro‐materials as an emerging tool to enhance cancer immunotherapy, and it comprehensively summarizes the different immunotherapeutic strategies addressed to date by using MSN.

## Graphical abstract

Considering the increasing potential of cancer immunotherapy and the great attention to mesoporous silica materials as drug delivery systems, numerous silica‐based immunotherapies have been developed. This review explores the role of mesoporous silica materials as an emerging tool to enhance cancer immunotherapy by describing the different developed scaffolds alongside the immunotherapeutic applications while discussing the benefits of nanomaterials.

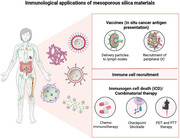

## 1 Introduction to Immunotherapy

Immunotherapy has emerged as a powerful useful approach for cancer treatment in recent years. Some of its advantages over traditional cancer therapies (i.e., chemotherapy, radiotherapy, and surgery) are the huge improvement in patients’ quality of life and survival percentage. Yet despite such progress, some drawbacks should be overcome, such as dose‐limiting toxicity, and low patient response. In this scenario, the possibility of combining immunotherapy with nanomedicine to enhance the therapeutic effect while minimizing side effects could soon result in immune oncology revolution.^[^ ^1^, ^2^ ^]^

**Figure 1:**
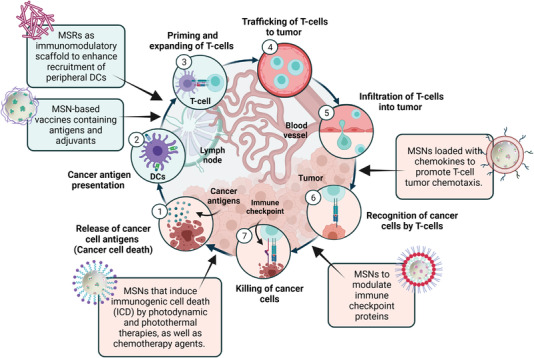
The cancer immunity cycle and the key points where mesoporous silica materials can be used. Adapted with permission.^[^ ^3^ ^]^ Copyright 2013, Cell Press.

Nano/micro‐immunotherapies can act on different aspects in the cancer immunity cycle (**Figure** **1**). Briefly, the cancer immunity cycle can be summarized in the following main steps: first, dying cancer cells release new antigens (neoantigens) to the tumoral microenvironment. Neoantigens are captured and processed by antigen‐presenting cells (APCs), which expose them to their major histocompatibility complexes (MHC‐I and MHC‐II). Then these peptides are presented in lymphoid organs to T‐cells through their CD4^+^ receptors, which results in T‐cell priming and activation to respond to the shown neoantigens. The activated T‐cells migrate and infiltrate the tumor bed, recognize cancer cells as foreign bodies and bind to them through the interaction of the T‐cell receptor (TCR) with its cognate antigen attached to MHC‐I. Finally, cytotoxic T‐cells eradicate malignant cells by means of a process named immunological cell death (ICD). This contributes to the release of more neoantigens to, thus, amplify the cycle.^[^ ^3^, ^4^, ^5^ ^]^ The next section explains the cancer immunity cycle in more detail. In addition, the cancer immunity cycle includes the integration of a large number of stimulatory and principally inhibitory regulators in each step (**Table** **1**).

**Table 1:** Description of the immune cells involved in anti‐ and pro‐tumorigenic processes

Immune cells	Role in tumor	Immune mediators secreted
Innate immune cells		
Macrophages		
M1 type	Pro‐inflammatory. Driven by LPS and IFN‐*γ* Elimination of cancer cells.	IFN‐*γ*, IL‐1, IL‐6, IL‐12, IL‐23, TNF‐*α*.
M2 type	Anti‐inflammatory. Driven by IL‐4, IL‐3. Stimulation of angiogenesis, lymphangiogenesis, cancer cell proliferation, epithelial‐mesenchymal transition, and immunosuppression.	IL‐10, TGF‐*β*, PGE2, EGF, VEGF, MMPs.
Natural Killers (NK)	Tumor cells killing directly or by secretion of cytokines.	Perforin, granzyme, TNF‐*α*.
Bridge between innate and adaptive immunity		
Antigen‐presenting cells (APC)	Presentation of endogenous and exogenous antigens to T‐cells by MHC molecules. The commonest cell type is dendritic cells (DC).	IL‐4, IL‐12, IL‐18.
Adaptive immune cells		
T‐cells		
CD8^+^	Differentiation to cytotoxic T‐lymphocytes (CTL). Direct killing of cancer cells.	Perforin, granzyme.
Helper CD4^+^ (Th1)	Secretion of pro‐inflammatory cytokines.	IL‐2, TNF‐*α*, IFN‐*γ*.
Regulatory CD4^+^ (Tregs)	Suppression of the priming, activation, and cytotoxicity of other immune cells, such as CTL, helper CD4+, macrophages, NK and neutrophils.	Contact‐dependent: PDL‐1, lymphocyte‐activation gene 3 (LAG‐3), CD39‐73, CTLA4, PD1.
Contact‐independent: IL‐10, TGF‐*α*, PGE2, adenosine, galectin‐1.		
B‐cells		
Plasma cells	Tumor‐reactive antibody secretion.	Typically IgG.
Regulatory B‐cells	Different phenotypes. The role is not clear, but in overall immunosuppression induction.	IL‐10, TGF‐*β*.

Nanomaterials, like those based on mesoporous silica nanoparticles (MSNs) and mesoporous silica rods' (MSRs) scaffolds, attempt to trigger cancer immunity cycle activation while avoiding harming healthy cells. They can serve as vaccine adjuvants; vaccine vehicles for cancer antigens and pathogen‐associated molecular patterns delivery to APC; tools for T‐cell priming, expanding, trafficking, infiltration, and recognition of cancer cells; agents that cause the release of tumor antigens by photodynamic and photothermal therapies; chemotherapy agents that cause the direct killing of malignant cells or inhibit the immune checkpoint; agents for starvation therapies or a combination thereof.

### 1.1 Main Strategies in Cancer Immunotherapy

#### 1.1.1 Cancer Cell Antigen Release and Presentation

The cancer immunity cycle is initiated by the release of the neoantigens generated due to the oncogenesis process. Chemotherapy, radiation therapy, photodynamic therapy (PDT), and photothermal therapy (PTT) can induce ICD, a type of cellular demise that triggers the release of tumor‐associated antigens (TAA) by stimulating a tumor‐specific immune response. The key parameters of ICD are: 1) exposure of “eat‐me” signals for dendritic cells (DCs); 2) secretion of ATP, a potent chemotactic signal for macrophage and DC precursors; and 3) the release of end‐stage degradation products that bind to the surface of DCs and allow optimal antigen processing and presentation. The capacity of ICD inducers, either alone or in combination with other immunotherapies, to produce potent innate and adaptive responses, has been tested in preclinical cancer models and clinical trials.^[^ ^6^, ^7^ ^]^

After the release of neoantigens by ICD, the next step is their internalization (phagocytosis or receptor‐mediated endocytosis) by professional APC: DC, B‐cells, and macrophages. These cells capture and process neoantigens by exposing them in their membrane through MHC‐I or MHC‐II to be recognized by T‐cells. The endogenous antigens presented in MHC‐I are generally recognized by TCR of CD8^+^ T‐cells, while exogenous antigens presented in MHC‐II are recognized by TCR of CD4^+^ T‐cells. However, APC can also present foreign antigens to the CD8^+^ T‐cells in MHC‐I during a process known as cross‐presentation. In this case, the interaction with CD26 present in the T‐cells with B7 costimulatory molecules (i.e., cluster of differentiation 80 [CD80] and cluster of differentiation 86 [CD86]) expressed on the surface of APC is required. Besides, APC express pattern recognition receptors as toll‐like receptors (TLR) in their membrane.^[^ ^8^ ^]^

Cancer cells develop immune‐evasive mechanisms, such as alterations in MHC expression or presentation,^[^ ^9^ ^]^ alterations in the expression of costimulatory molecules,^[^ ^10^ ^]^ or the attachment of CD80 and CD86 to cytotoxic T‐lymphocyte‐associated protein 4 (CTLA‐4) (inhibitory molecule) instead of to CD28 in activated T‐cells.^[^ ^11^ ^]^ In addition, some tumor cells are capable of acquiring APC properties due to the aberrant expression of costimulatory molecules, and professional APC can turn out malignant and maintain their properties as tumor B‐cells in lymphomas.^[^ ^12^ ^]^ Several cancer therapies are directed to reinforce tumor antigen presentation by the delivery of peptidic or DNA vaccines and TLR agonists.^[^ ^13^ ^]^ The FDA has approved three oncovaccines:^[^ ^14^ ^]^ BCG,^[^ ^15^ ^]^ Sipuleucel‐T,^[^ ^16^ ^]^ and IMLYGIC or T‐VEC^[^ ^17^ ^]^ (**Table** **2**).

**Table 2:** Summary of immunotherapy‐based strategies being developed and used in trials

Strategy	Compounds	Basis and application	FDA approval
1. Cancer cell antigen release and presentation	BCG	Attenuated *Mycobacterium bovis* for urothelial carcinoma.	Yes
	Sipuleucel‐T	APC stimulated with prostatic acid phosphatase linked with GM‐CSF for castration‐resistant prostate cancer.	Yes
	IMLYGIC/T‐VEC	Attenuated oncolytic virus (herpes 1‐based) for melanoma.	Yes
2. Priming and expanding T‐cells	Ipilimumab	Anti‐CTLA‐4 mAb for melanoma.	Yes
	CDX	Agonist anti‐CD27 mAb in clinical trials.	No
	Urelumab and PFZ‐05082566	Agonist anti‐CD137 mAb in clinical trials.	No
	MEDI6383 and MOXR0916	Agonist humanized anti‐OX40 mAbs in clinical trials.	No
	IL‐2	Recombinant IL‐2 for renal cancer and metastatic melanoma.	Yes
3. T‐cell trafficking and infiltration	Bevacizumab	Anti‐VEGF mAb for different cancer types.	Yes
4. Recognition of cancer cells by T‐cells	Axicabtagene ciloleucel	CAR‐T therapy for diffuse large B‐cell lymphoma.	Yes
	Tisagenlecleucel	CAR‐T therapy for diffuse large B‐cell lymphoma and acute lymphoblastic leukemia	Yes
	Catumaxomab	BiTEs therapy. Anti‐EpCAM (epithelial cell adhesion molecule) mAb for carcinomas.	Yes
	Blinatumomab	BiTEs therapy. Anti‐CD19 mAb for acute lymphoblastic leukemias.	Yes
5. Killing cancer cells	Nivolumab and Pembrolizumab	Anti‐PD‐1 mAb for melanoma and metastatic cervical cancer.	Yes
	CA‐170	Anti‐VISTA (v‐domain Ig suppressor of T‐cell activation) mAb for lymphomas.	No
	NCT02061761	Anti‐LAG‐3 mAb for refractory hematologic malignancies.	No
	TSR‐022	Anti‐TIM‐3 (T‐cell immunoglobulin and mucin 3) mAb for advanced solid tumors.	No
	OMP‐31M32 and BMS‐986207	Anti‐TIGIT (T‐cell immunoglobulin and immunoreceptor tyrosine‐based inhibitory domain) mAb for advanced metastatic solid tumors.	No
	CB‐1158	Small molecule inhibitor of arginase for advanced solid tumors.	No
	Epacadostat	Small molecule inhibitor of IDO‐1 for melanoma.	No
	GC1008	Anti‐TGF‐*β* mAb for advanced malignant melanoma.	No
	Galunisertib	Anti‐TGFR‐*β* mAb for hepatocellular carcinoma.	No
	M7824	Chimeric Anti‐PD‐1 mAb and TGF‐*β* receptor	No

#### 1.1.2 Priming and Expanding T‐Cells

DC stimulates naïve T‐cells by the cross‐presentation of cancer antigens in lymphoid organs, which results in the priming and expanding of effector or memory T‐cells. CD8^+^ cytotoxic T‐lymphocytes (CTL) are the key immune cells for killing cancer cells, while the CD4^+^ T helpers (Th) cells population, which includes cells Th1, Th2, Th17, and regulatory T‐cells (Tregs), plays the role of orchestrating the immune response.^[^ ^18^ ^]^ The effectiveness of T‐cells activation depends on TCR, co‐stimulatory molecules, and cytokine stimulation. Different strategies based on the modulation of these factors have been developed.^[^ ^19^ ^]^ One approach is to use immune checkpoint inhibitors, for instance the antibodies addressed to cytotoxic T‐lymphocyte antigen‐4 (CTLA‐4) like Ipilimumab. In contrast, another strategy is based on the activation of co‐stimulatory receptors like CD27, CD137, and OX40 with agonist antibodies, such as Urelumab or polynucleotide‐based aptamers.^[^ ^20^ ^]^ Additionally, the administration of immunostimulatory cytokines like recombinant interleukin‐2 (IL‐2) has also demonstrated anticancer activity via T‐cell activation and expansion in preclinical and clinical studies (Table 2).^[^ ^21^ ^]^

#### 1.1.3 T‐Cell Trafficking and Infiltration

Activated effector T‐cells circulate through the bloodstream from lymph nodes to tumors where they infiltrate. Several factors are important in this step, including the appropriate match between the tumor‐secreted chemokines and receptors expressed in T‐cells, the presence of an aberrant vasculature, and interactions between TCRs and adhesion molecules on endothelial cells (EC).^[^ ^22^ ^]^ Bevacizumab, an antibody that blocks the vascular endothelial growth factor (VEGF) secreted by tumors, is a promising candidate to enhance the infiltration of lymphocytes by promoting “vascular normalization” and EC activation (Table 2).^[^ ^23^, ^24^ ^]^

#### 1.1.4 Recognition of Cancer Cells by T‐Cells

After having infiltrated the tumor, T‐cells specifically recognize and bind to cancer cells through the interaction between the TCR receptor and the tumor antigen bound to MHC‐I. In cancer patients, tumor antigens may not be detected; T‐cells may recognize tumor antigens as self and, therefore, create T regulatory cell responses rather than effector responses. The challenge of cancer immunotherapy in this step lies in reinitiating a self‐sustaining cancer immunity cycle to enable T‐cells to recognize tumors.^[^ ^25^ ^]^ Two main approaches for T‐cell redirection involve their genetic modification with chimeric antigen receptors (CAR), like Axicabtagene ciloleucel and Tisagenlecleucel, or the use of recombinant proteins designated bispecific T‐cell engagers (BiTes), such as Catumaxomab and Blinatumomab (Table 2.)^[^ ^26^, ^27^, ^28^, ^29^, ^30^, ^31^ ^]^

#### 1.1.5 Killing Cancer Cells

If the cancer immunity cycle proceeds until T‐cells recognize, but fail to kill cancer cells, several therapies can act in this step. The most widely explored immunotherapy class is immune checkpoint inhibition, commonly mediated by blocking the programmed cell death protein 1 (PD‐1)/programmed death‐ligand 1 (PD‐L1) interaction, which suppresses T‐cell activity (see below). Moreover, other approaches include the inhibition of the enzymes that reduce T‐cell activity, such as indoleamine 2,3‐dioxygenase (IDO) or arginase, as well as the suppression of transforming growth factor beta (TGF‐*β*) secreted by tumors (Table 2).^[^ ^25^, ^32^, ^33^, ^34^ ^]^

Immune checkpoints normally maintain appropriate immune responses and protect healthy tissue from immune attack.^[^ ^27^ ^]^ When T‐cells are activated, they express PD‐1, which binds to its ligands and negatively regulates effector T‐cell activity. To evade recognition and elimination by T‐cells, tumor cells express this ligand (PD‐L1) by generating an immunosuppressive effect. FDA approved therapies using monoclonal antibodies to prevent those interactions and, therefore, stimulate antitumor immunity (Table 2). In addition, many trials involving checkpoint inhibitors in combination with other agents are ongoing (>700).^[^ ^27^, ^28^, ^32^, ^33^, ^34^, ^35^ ^]^

Besides, tumor cells secrete several immunosuppressive factors in the tumor extracellular matrix (TME). Targeting these factors may reactivate cells for antitumor immunity (Table 2). IDO and arginase inhibitors are being tested in clinical trials with patients, for example, CB‐1158.^[^ ^36^, ^37^, ^38^ ^]^ TGF‐*β* is produced in large amounts in the TME by leukocytes and is crucial for controlling the development of excessive immune responses and for maintaining immune homeostasis.^[^ ^27^, ^35^ ^]^ In the premalignant cells phase, TGF‐*β* promotes immunological tolerance by directly suppressing the cytolytic activity of NK and CTL. However, in a late cancer development stage, high TGF‐*β* levels stimulate tumor progression via effects on the stroma. TGF‐*β* inhibition has been studied using different strategies, such as antisense oligonucleotides, TGF‐*β*‐neutralizing antibodies, and TGF‐*β* receptor kinase inhibitors (Table 2).^[^ ^27^, ^39^, ^40^ ^]^

### 1.2 Problems and Limitations

Many of the immune treatments commented above have reached preclinical studies, but translation into patients remains challenging and raises concerns, including the development of autoimmune toxicities, the targeting of homeostatic functions like angiogenesis and the risk of developing new malignancies.^[^ ^35^ ^]^ Amplifying the entire cycle may provide anticancer activity, but at the potential cost of undesired damage to normal tissue.^[^ ^5^, ^25^ ^]^ Similarly, response rates to immunotherapy are still modest because of the complexity of immune‐tumor interactions and the existence of redundant mechanisms of tumor‐mediated immune suppression.^[^ ^27^ ^]^ Cytokines, checkpoint inhibitors and agonistic antibodies have similar limitations and challenges. They produce substantial autoimmunity and adverse effects, which limit the allowed dose. However, given their short half‐life, effective treatments require high‐dose injections that cause vascular leakage and cytokine release syndrome. Thus the main goal in developing these therapies is to achieve a targeted and/or controlled release of immunotherapeutics that would minimize off‐target effects.^[^ ^27^, ^30^ ^]^ The last immunotherapy strategy that ended in clinical practice, that is, the chimeric antigen receptor T‐cell (CAR‐T) therapy, also encountered some problems, such as possible target antigen loss on tumor cells, low CAR‐T cell levels reaching tumors, their suppression by negative regulators in the TME, and acquired resistance, which all limit this new strategy. More efforts are required to improve these treatments. Understanding the fundamentals of tumor antigen production and maintenance, antigenic evolution, and tumor immune heterogeneity is essential. The development of predictive biomarkers or personalized immunotherapy could help to overcome some of these limitations. Additionally, delivery technologies are very promising and can, therefore, be used to modulate immunogenicity in cold tumors, reduce systemic toxicity, and deliver combinations of therapeutics.^[^ ^27^ ^]^

## 2 Role of Nanotechnology

Despite immunotherapy having emerged as a potential strategy to fight cancer, the efficacy of some immunotherapeutic agents is still limited.^[^ ^41^, ^42^ ^]^ To overcome these drawbacks, nanotechnology has been pointed out as a promising alternative capable of improving both efficacy and safety in cancer immunotherapy.^[^ ^43^, ^44^ ^]^ In fact, cancer therapy has benefited the most from nanotechnology approaches. For a few years now, nanoimmunotherapies have been developed using liposomes,^[^ ^45^, ^46^ ^]^ polymers,^[^ ^47^ ^]^ gold nanoparticles (NPs),^[^ ^48^ ^]^ MSN,^[^ ^49^ ^]^ and other nanomaterials.^[^ ^50^, ^51^ ^]^ In general, NPs act by protecting immunotherapeutic agents (antibodies, antigens, cytokines, small molecules, etc.) through blood circulation to the target site.^[^ ^52^, ^53^ ^]^ Nanomaterials are also considered good candidates for immunotherapy given their intrinsic ability to target lymph nodes, immune cells as well as tumors.^[^ ^54^, ^55^, ^56^ ^]^ Besides, specific targeting ligands can be used to enhance the delivery of immunotherapeutic agents. In this way, NPs can help to achieve a more controllable immune response through selective cargo delivery and can limit unsought immune overreaction or systemic toxic effects from some immunotherapeutic agents (i.e., cytokines).^[^ ^57^, ^58^ ^]^ NPs can also enhance the immunostimulatory effect by helping to achieve a suitable immune response. Indeed, a potential advantage of NPs to be exploited in immunotherapy is their intrinsic immunogenicity. Cationic and small‐sized NPs are easily internalized by some APC types like macrophages and DC, which makes them suitable for cancer vaccine development.^[^ ^59^, ^60^, ^61^ ^]^ Bearing in mind the increasing demand for combinatorial therapies to improve the effectiveness of cancer immunotherapy, another key advantage of using nanomaterials is the possibility of combining different therapeutic strategies in the same nanocarrier. PTT, PDT, and conventional chemotherapy can be effectively combined with immunotherapy to achieve synergistic therapy in cancer treatment.^[^ ^53^ ^]^

Of the different available nanomaterials, MSN are promising candidates for cancer immunotherapy due to their unique properties. In the last few years, attention has been paid to MSN as drug delivery systems in the biomedical field,^[^ ^62^, ^63^, ^64^ ^]^ although MSN have also been used for sensing^[^ ^65^, ^66^ ^]^ and interparticle communication protocols.^[^ ^67^, ^68^, ^69^, ^70^ ^]^ Their large surface area and high loading capacity make MSN suitable for the delivery of a wide variety of drugs, antibodies, genes, proteins, and peptides.^[^ ^71^, ^72^ ^]^ It is noteworthy that their easy surface modification allows the design of gated materials to release entrapped cargo upon the application of a selected stimulus.^[^ ^73^, ^74^, ^75^ ^]^ In these gated materials, MSN are loaded with a certain payload, and biomolecules or supramolecular ensembles are attached to the external surface that act as gatekeepers (also known as molecular gates or nanovalves). Following this design, the cargo is retained inside the porous structure and is delivered only at will in the presence of a selected stimulus. These unique chemical features are also a potential advantage for combining synergistic therapies because MSN can hold different biomolecules and other NPs.^[^ ^76^, ^77^ ^]^ Besides, and especially for immunotherapeutic approaches, MSN can act as adjuvants to promote immune cell recruitment and activation.^[^ ^78^, ^79^ ^]^ Bearing all this in mind, numerous silica‐based immunotherapies have been developed in only a few years and an increasing number of examples is expected to be published in the near future. Below, a comprehensive overview of mesoporous silica‐based immunotherapies is presented, including cancer vaccine development, immune cell recruitment, and MSN‐based systems for the ICD of cancer cells and the design of synergistic therapies.

## 3 Mesoporous Silica‐Based Materials Applications

### 3.1 Vaccines (In Situ Cancer Antigen Presentation)

#### 3.1.1 Delivery Particles to DC and Lymph Nodes. MSN as Adjuvants and Antigen Carriers

The ability of mesoporous silica‐based nanomaterials to act as adjuvants, an immunological agent capable of activating APC and, thus, eliciting an immune response, has been described in recent years. Compared to traditional adjuvants, these nanomaterials can induce both tumoral and cell‐mediated immune responses. They can also be employed as nanocarriers for antigen delivery in cancer vaccination.^[^ ^80^ ^]^ The rational design of potential mesoporous silica nanomaterials‐based vaccines is guided by their structural parameters, such as morphology, porous structure, and functional groups on the surface.

In the beginning, the different approaches employed plain MSN or with a few modifications like aminated MSN as a delivery tool of immune adjuvants. The commonest adjuvant is cytosine–phosphate–guanosine oligodeoxynucleotides (CpG ODNs), which target toll‐like receptors 9. For example, Tao et al. developed biocompatible aminated MSN (MSN‐NH_2_) to load CpG ODN to prevent degradation from serum nucleases and to achieve greater immune stimulation.^[^ ^81^ ^]^ Following the same objective, Zhang et al. developed boron nitride nanospheres (BNN) functionalized with mesoporous‐silica (MS) and amine groups (BNNS@MS‐NH_2_/CpG ODN).^[^ ^82^ ^]^ This nanodevice displayed a similar behavior as that described before, but with improved features, good biocompatibility, and enhanced internalization to, thus, achieve a greater induction of the immune response. Besides, the incorporation of an iron magnetic core into the mesoporous silica scaffold described in other work by Zheng et al. is an advantage over other nanodevices by allowing magnetic guidance toward the target and, thus, enhancing an immune effect and tumor suppression.^[^ ^83^ ^]^

Furthermore, the capacity of different porous silica scaffolds to serve as carriers of antigens has also been studied, and the commonest antigen model is ovalbumin (OVA). Wang and co‐workers loaded MSN with different antigens, such as OVA, cancer cell fragments of Lewis lung carcinoma (LLC) or homogenized autologous LLC cancer tissue to evaluate their efficacy in preventive and therapeutic animal models.^[^ ^84^ ^]^ In all cases, antigen‐loaded MSN exhibited remarkable anticancer immunity. In the preventive model, the immunized group with NPs exhibited a significant reduction in tumor size and a remarkable percentage of cancer‐free mice (50–60%). In the therapeutic model, both the CD4^+^ and CD8^+^ T‐cell populations were enhanced in all the immune organs. In addition, C57BL/6 mice of different ages (6‐ or 18‐week‐old) were immunized with OVA combined with alum or MSN. The results indicated an enhanced secretion of interferon‐*γ* (IFN‐*γ*) and IL‐2, typical of Th1 responses (*≈*6‐ and 2‐fold higher, respectively, than in the control groups), plus a respective 25‐ and 5‐fold increase in the typical interleukin‐4 (IL‐4) and interleukin‐10 (IL‐10) levels for Th2 responses in the groups treated with NPs. The results also showed the effectiveness of MSN to emphasize Th1 and Th2 immunity in both young and elder mice. Finally, with the same model, it was found that MSN significantly improved titers immunoglobulin A, immunoglobulin G2a (IgG2a), immunoglobulin G (IgG), and immunoglobulin G1 (IgG1) compared to controls, mostly 38 days after the first vaccine injection.

**Figure 2:**
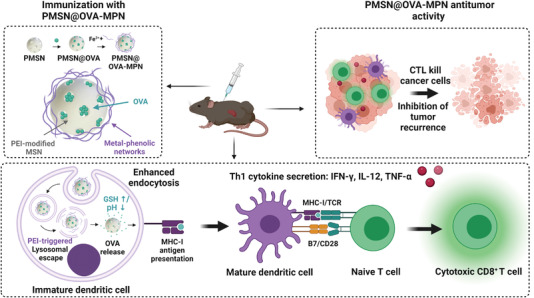
Illustration of the PMSN@OVA‐MPN synthesis procedure and anticancer performance. Immunization with the nanodevice induced Th1 cytokine secretion, DC maturation and, thus, cytotoxic CD8^+^ T‐cell activation, which resulted in toxicity against tumor cells. Adapted with permission.^[^ ^85^ ^]^ Copyright 2020, ACS.

In parallel, Zhou et al. engineered a multifunctional cancer nanovaccine composed of a MSN functionalized with polyethyleneimine (PEI) (PMSN), loaded with the model antigen OVA and coated by disulfide‐bond‐involved metal‐phenolic networks (MPN) (PMSN@OVA‐MPN) (**Figure** **2**).^[^ ^85^ ^]^ Significant OVA delivery (60% and 80% in 10 min, respectively), and efficient lysosome escape due to the PEI‐triggered proton sponge effect, thus, allowed cytosolic OVA release and the subsequent antigen cross‐presentation by MHC‐I to be achieved. For antitumor studies, mice (C57BL/6) were inoculated with E.G7‐OVA lymphoma cells by subcutaneous injection in the right flank before immunization with PMSN@OVA‐MPN. For the tumor prophylaxis study, immunization was done previously to cancer cell inoculation. An increased expression of the co‐stimulatory factor CD86 and MHC‐I in lymphocytes (20% and 60% rise over free OVA) from the mice immunized with PMSN@OVA‐MPN was noted. High secretion of the typical cellular immunity cytokines, IFN‐*γ*, interleukin‐12 (IL‐12) and tumor necrosis factor alpha (TNF‐*α*), and also of an OVA‐specific IgG antibody, an indicator of Th1‐biased cellular immunity, took place in spleen lymphocytes. An increased percentage of CD8^+^ T‐cells and effector T‐memory cells was obtained for splenocytes compared to the controls (10% higher). Finally, the PMSN@OVA‐MPN vaccine manifested the best tumor inhibition effect in both the tumor volume analysis and survival curves (100% until day 33), as well as the greatest antitumor recurrence.

In addition to the widely used antigen OVA, other authors have developed MSN‐based vaccines using other immune‐stimulatory molecules. Li et al. have reported a mesoporous silica/calcium phosphate (CaP) composite loaded with tuberculin‐purified protein derivative (PPD) (PPD‐MS/CaP) as an effective adjuvant for cancer immunotherapy.^[^ ^86^ ^]^ In vitro studies have demonstrated that PPD‐MS/CaP stimulates macrophages more effectively than PPD and PPD‐MS according to the granulocyte‐macrophages colony‐stimulating factor (GM‐CSF) levels (6 vs 2 pg mL^−1^, respectively). LLC cells were injected subcutaneously into the left flank of mice. After 10 days, TTA were obtained from tumor tissue. Different NPs were mixed with TTA and injected into the original tumor site three times. Tumor recurrence was monitored for 28 days. Then live LLC cells were injected into mice in the right flank. Tumor growth was monitored for 35 days. The tumors on the left side were significantly reduced by PPD‐MS/Cap treatment. In the right flank, only 50% of the mice treated with PPD‐MS/Cap developed tumors after 11 days compared to the PPD‐MS and free PPD groups in which 75% and 80% of mice developed tumors after the same time, respectively.

Given the role of the chemical surface properties of nanomaterials for triggering and modulating an immune response, Yang et al. used MSR with different modifications.^[^ ^87^ ^]^ The surface of MSR was decorated with amino moieties (MSRs‐NH_2_) octadecyl trimethoxysilane, to generate a hydrophobic material (MSNR‐C18), or nondecorated (MSNR‐OH), and the materials were additionally loaded with the model antigen OVA. Different OVA loading capability and delivery was observed for the three solids (250, 400, and 550 mg g^−1^ of protein loading, respectively). Subcellular localization and enhanced internalization were revealed for MSRs‐NH_2_ (endo/lyso‐somes) and MSRs‐C18 (cytosol). MSRs‐C18 was the most efficient vaccine and induced CD86 activation in APC, with subsequent cytokine IFN‐*γ* secretion (up to 600 pg mL^−1^) and splenocyte proliferation in the immunized C57BL/6 mice. The authors demonstrated that the surface chemistry of MSR plays a crucial role in modulating the immune response. Whereas —NH_2_ provoked a bias toward T‐helper 2 immunity, the hydrophobic‐octadecyl modification enhanced the antigen uptake by APC, which was facilitated by endosomal escape and, thus, achieved greater maturation of both DC and macrophages in ex vivo assays.

According to described works above, the most basic vaccines are composed of plain MSN or slightly modified (mainly aminated) ones loaded with adjuvants or antigens. These kinds of materials exhibit good biocompatibility and prevent cargo from nuclease serum degradation. As a result, cargo bioavailability is enhanced, and thus, the secretion of the typical cytokines associated with macrophage activation and Th1 and Th2 responses is achieved. Later other alternatives with a higher load capacity, such as hollow mesoporous silica nanoparticles (HMSNs), were implemented. For instance, HMSN were compared to typical MSN by Wang et al. For that purpose, fluorescein‐conjugated OVA (F‐OVA) as the model antigen and poly(I:C) (PIC) as immunopotentiator were absorbed in HMSN yielding (HMS‐OVA‐Po).^[^ ^88^ ^]^ MSN loaded with F‐OVA and PIC (MS‐OVA‐Po) were also prepared as controls. Anticancer immunization was evaluated in a lymphoma E.G7‐OVA cancer model. While the mice treated with saline‐OVA developed cancer with a large tumor size, the mice immunized with HMS‐OVA‐Po and MS‐OVA‐Po presented smaller tumor sizes, with 60% and 80% of the cancer‐free mice at the endpoint. The mice immunized with HMS‐OVA‐Po and MS‐OVA‐Po did not show metastasized cancer cells to lymph nodes. Besides, the mechanism of cancer immunity was characterized, and the results demonstrated a higher level of the CD4^+^ and CD8^+^ T‐cell populations in the mice treated with HMS‐OVA‐Po compared to the immunized mice with MS‐OVA‐Po and saline‐OVA (10% added). The increased immunization achieved by HMS‐OVA‐Po was attributed to the better loading capacity of the NPs with large cavities.

Wang and co‐workers evaluated the ability of HMSN to carry and deliver a selected payload.^[^ ^78^ ^]^ To do so, they used three model cargoes: ferritin, F‐OVA, and LLC tumor antigen. OVA induced Th1‐ (IFN‐*γ* and IL‐2) and Th2‐ (IL‐4 and IL‐10) specific cytokine secretion to serum in comparison to the controls, alum, and no adjuvants. In vivo studies have shown marked tumor growth inhibition in the mice immunized with LLC‐HMSN and a stronger induction of CD4^+^ and CD8^+^ effector memory T‐cell populations in bone marrow after 2 months (50% vs 30% in the controls). The study demonstrated that HMSN can induce a potent cellular antitumor immune response, including enhanced CD4^+^ and CD8^+^ T‐cell populations and Th1 and Th2 immunity in vivo.

**Figure 3:**
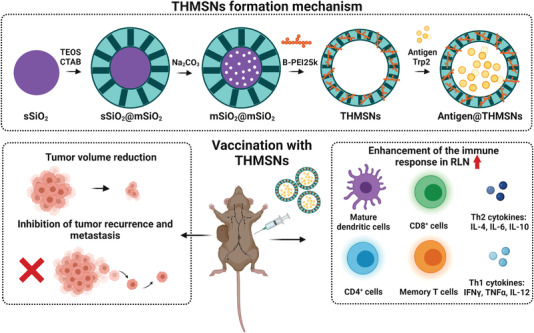
Scheme for THMSN preparation and cancer vaccination. THMSN bring about a strong immune response in regional lymph nodes (RLN) with cytokine secretion, maturation of immune cells, tumor volume reduction, inhibition of tumor recurrence, and metastasis. Adapted with permission.^[^ ^89^ ^]^ Copyright 2019, ACS.

To improve the anticancer immune response, the incorporation of polymeric or lipidic material into hollow NPs has been evaluated. Liu et al. demonstrated the potential of PEI incorporated into thin‐shell hollow MSM (THMSN) (**Figure** **3**).^[^ ^89^ ^]^ To prepare nanodevices, solid silica NPs were synthesized and coated with a mesoporous silica shell and modified through PEI etching to obtain THMSN. Finally, THMSN were loaded with a melanoma‐derived antigen peptide (Trp2) to yield the final cancer vaccine Trp2@THMSNs. To evaluate the antitumor effect of Trp2‐related vaccines, C57BL/6 mice were immunized three times and later injected with B16‐F10 cells. The results showed that the group vaccinated with Trp2@THSMSs reached the smallest tumor volume (250 vs 2000 mm^3^ in the control), the greatest inhibition of tumor occurrence, and the most prolonged survival rate compared to the controls. In second place, a rechallenge tumor model was used to investigate the potential long‐lasting effects of vaccination. For this purpose, TAA mixed with THMSN were subcutaneously injected into mice three times. The TAA‐THMSN vaccine showed the most enhanced antitumor activity compared to the controls, with delayed tumor development in both flanks and triggering of immunological memory, detected as the percentage of CD4^+^ T and CD8^+^ T‐cells in bone marrow (30% and 40%, respectively).

**Figure 4:**
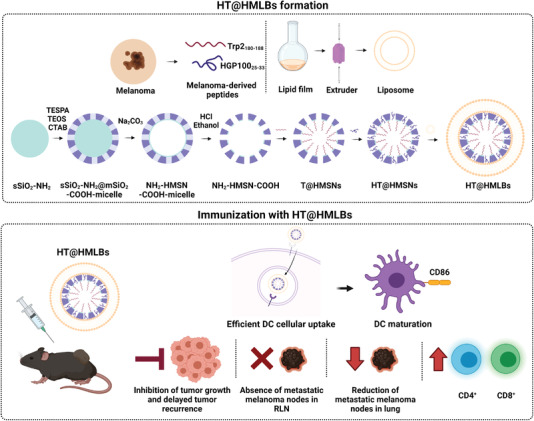
HT@HMLBs for dual melanoma antigen delivery. Representation of their synthesis and immune capabilities, DC maturation, lymphocyte proliferation, and inhibition of tumor growth and tumor recurrence. Adapted with permission.^[^ ^90^ ^]^ Copyright 2017, Wiley.

Similarly, Xie et al. have reported HMSN coated with a lipid bilayer (DOPC: cholesterol: DOPE‐PEG2000) (HMLBs) and monophosphoryl lipid A to load two melanoma‐derived antigen peptides: hydrophobic TRP2 and hydrophilic HGP100 peptides (HT@HMLBs) (**Figure** **4**).^[^ ^90^ ^]^ These authors indicated the sustained release of TRP2 and HGP100 peptides (36% and 47% in 168 h, respectively) and efficient cellular uptake in bone marrow‐derived dendritic cells (BMDC). An enhanced antitumor immune response was significantly induced by HT@HMLBs in C57BL/6 mice and a higher DC maturation level was achieved (40% increase in DCs‐CD86 surface expression), as was the activation of CD4^+^ and CD8^+^ T‐lymphocytes with marked tumor‐killing capability (12% increase in CD107a^+^ expression). In addition, HT@HMLB delayed tumor occurrence (19 vs 11 days) and inhibited tumor growth in C57BL/6 mice compared to the controls without MLPA in melanoma and lung metastatic models. A remarkable reduction of lung metastatic nodules with smaller size (15 nodules) was found in the vaccinated mice with NPs compared to control groups (≈90 nodules).

**Figure 5:**
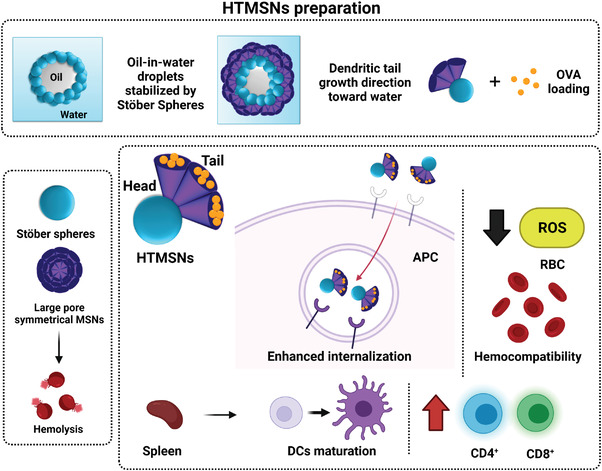
Hemocompatible HTMSN as a cancer vaccine vehicle to induce lymphocyte and DC maturation. Adapted with permission.^[^ ^91^ ^]^ Copyright 2017, ACS.

The efficacy of other high‐loading‐capacity nanomaterials to act as a vaccine has also been investigated. Customized head–tail‐structure asymmetric MSN (HTMSN) have been described by Abbaraju et al. (**Figure** **5**).^[^ ^91^ ^]^ HTMSN were composed of solid or porous head particles attached to dendritic tails with large mesopores (11–28 nm). In vitro hemolytic studies demonstrated that asymmetrical HTMSN gave the lowest hemolysis percentage in a dose‐dependent manner compared to other mesoporous silica scaffolds. Besides, the internalization of the HTMSN labeled with fluorescein isothiocyanate by APCs in mice isolated spleen DC and macrophages was significantly higher in the asymmetrical nanodevices. To examine APC maturation, nanodevices were loaded with OVA and incubated with mice‐isolated splenocytes. The results showed greater T‐cell population (CD40 and CD86 molecule expression) induction when asymmetric nanoparticles (HTMSN) were used compared to the controls.

To increase the possibilities of the mesoporous silica materials as vaccines, Lee et al. developed hollow MSN with extra‐large mesopores (H‐XL‐MSNs) capable of the high loading of model proteins and adjuvants.^[^ ^92^ ^]^ H‐XL‐MSN were decorated on the surface with both the immune adjuvant PEI and the model antigen OVA. The PEI‐coated H‐XL‐MSN showed good biodegradability properties in acidified simulated body fluid with degradation in 6 days and high cellular uptake in BMDC (55% vs 10% of free PEI). Besides, the incubation of BMDC with the OVA‐loaded PEI‐coated H‐XL‐MSN induced their maturation. In vivo studies in melanoma tumor‐bearing mice have indicated that PEI‐modified H‐XL‐MSN loaded with OVA vaccination generated twofold higher levels of antigen‐specific CTL and the highest tumor suppression and survival rate (30 days) compared to the NP without PEI and free OVA.

These studies above, demonstrated that HMSN and high‐load‐capacity MSN allow the release of a larger amount of cargos. As a result, a greater immune response is obtained. Particularly, in vivo studies demonstrated that this kind of NPs induces high tumor growth inhibition. Immunological memory was also evaluated and showed delayed or inhibited tumor development, as well as effector memory T‐cells.

The potential of dendritic materials has also been analyzed in the work by Yang et al.^[^ ^93^ ^]^ They designed multishelled dendritic mesoporous organosilica hollow spheres (DMOHS) with a controllable number of shells (S) to be used as adjuvants in cancer immunotherapy. DMOHS‐2S, DMOHS‐1S, and DMSHS‐2S were synthesized with two, one, and two shells, respectively. Organosilica NPs were employed with DMOHS‐2S and DMOHS‐1S, but DMSHS‐2S had a pure silica composition. DMOHS‐1S and DMOHS‐2S were loaded with the model antigens OVA or melanoma B16‐F10 tumor cells (tumor fragments [TF]) fragments. The data collected showed that DMOHS‐2S vaccination induced an improvement in both Th1 (IL‐12, IFN‐*γ*, and TNF‐*α* secretion) and Th2 immunity (IL‐4 secretion) in the splenocytes from the C57BL/6‐immunized mice versus the controls. These authors also observed delayed tumor occurrence (31 days), a reduction in tumor size, and an improved survival rate (50%) in the mice vaccinated with DMOHS‐2S after the B16‐F10 cells injection. Overall, the authors supported the benefits of using a double‐shelled organosilica composition for developing antitumor nanovaccines by considering that NPs' hydrophobicity and sustained antigens release were key factors for achieving an enhanced immune response.

The next step on the way to obtain an efficient vaccine is to combine both adjuvants and antigens in the same material, as well as safer scaffolds able to be easily degraded in the body. This was the case of Lu et al., who designed biodegradable GSH‐depletion mesoporous organosilica NPs functionalized with PEI and loaded with OVA and with CpG ODNs (GDMON‐P‐OVA‐CpG).^[^ ^94^ ^]^ Enhanced cellular uptake compared to the free antigens and successful endosomal escape were confirmed in RAW264.7 cells. A drop in the GSH levels, along with an increase in the ROS levels, was detected in the GDMON‐P‐OVA‐CpG‐vaccinated mice. Besides, GDMON‐P‐OVA‐CpG induced a potent CTL response in mice spleens after 7 post‐vaccination days compared to other groups. To evaluate tumor inhibition, mice were subcutaneously inoculated with B16‐OVA cells after being vaccinated twice. A significant reduction in tumor size was obtained with the GDMON‐P‐OVA‐CpG nanodevice. In the same way, Wang et al. developed plain mesoporous silica nanospheres doped with Ca, Mg, and Zn (MS‐Ca, MS‐Mg, and MS‐Zn) in which chicken egg OVA was adsorbed to act as an adjuvant.^[^ ^95^ ^]^ MS‐Ca, MS‐Mg, and MS‐Zn nanospheres showed 20% higher degradation rates than the undoped MS nanospheres in vitro and in vivo, and MS‐Zn obtained the highest degradation rate. To perform an in vivo anticancer test, C57BL/6J mice were subcutaneously injected three times with the corresponding adjuvant (Alum, MS, MS‐Ca, MS‐Mg or MS‐Zn nanospheres) mixed with OVA three times. After 14 days, mice were injected with E.G7‐OVA lymphoma cells in the right flank. The MS, MS‐Ca, MS‐Mg, and MS‐Zn nanospheres loaded with OVA showed marked inhibition of cancer growth and higher CD4^+^ and CD8^+^ T‐cell population levels, and the MS‐Zn‐OVA gave the highest percentage of CD4^+^ and CD8^+^ T‐cell activation (17.5% and 15%, respectively). The cells from the immunized mice draining lymph nodes (dLN) were co‐cultured with OVA for 3 days and an increase in Th1 (INF‐*γ*) and Th2‐type (IL‐4) cytokines secretion was detected. The OVA‐loaded MS‐Zn obtained the highest activation rate and the most IFN‐*γ* secretion (up to 300 vs 50 pg mL^−1^ with free OVA). In another interesting work, stellated fibrous MS nanospheres were used to adsorb PIC on their external surface as an immunopotentiator, and F‐OVA as an antigen‐specific cancer to E.G7‐OVA lymphoma cells, to yield MS‐OVA‐PIC.^[^ ^96^ ^]^ Cellular uptake demonstrated that the OVA‐loaded NPs were efficiently internalized by BMDC and promoted BMDC maturation in vitro, which gave a bigger CD11c^+^CD86^+^ cell population compared to the controls (sodium phosphate buffer [PBS]) (80% vs 53%). C57BL/J6 mice were treated with MS‐OVA‐PIC by subcutaneous injection three times. After 14 days, the E.G7‐OVA cells were injected into mice. The results showed that the mice immunized with MS‐OVA‐PIC achieved greater anticancer immunity than the control groups (OVA‐ and OVA‐PIC‐treated mice). Besides, the splenocyte analysis confirmed the presence of bigger CD4^+^ and CD8^+^ T‐cell populations and, thus, confirmed anticancer immunity.

Undoubtedly silica‐based nanomaterials with the appropriate cargo can induce a potent immune response (both in vivo and in vitro) and generate immunological memory. They have been demonstrated to be effective as vaccines and generate a greater immune response than free antigens/adjuvants. Among the different reviewed scaffolds, the dendritic materials offer the principal advantage of most of them being biodegradable as well as allowed the sustained release of antigens, which are key for an enhanced immune response.

#### 3.1.2 Recruitment of Peripheral DC to an Immunomodulatory Scaffold

Another approach to improve cancer immunotherapy is based on the development of injectable or implantable 3D materials for the spatio‐temporal modulation of immune cell populations, including DC and cytotoxic T‐cells. This strategy provides an in situ repository of the patient's own immune cells in the tumor that can promote and maintain robust and long‐lasting immune responses. In this context, biomaterials based on MSR assembly have recently stood out for their larger surface area and improved loading capacity for the development of immunomodulatory scaffolds.

The earliest study using MSRs scaffolds to recruit immune cells was conducted by Kim et al.^[^ ^97^ ^]^ They prepared bare high‐aspect‐ratio MSR (88 µm × 4.5 µm length and diameter) co‐loaded with GM‐CSF (a chemoattractant to immune DC), CpG‐ODN, and OVA to examine their potential as cancer vaccines. These authors demonstrated the ability of MSR to spontaneously form 3D platforms in vivo by observing the formation of a nodule (≈25 mm^3^) in female C57Bl/6J mice 4 h after their subcutaneous injection. An analysis of the host immune cells recruited to the scaffold over time showed a significant increase for MSRs with high‐aspect ratio compared to the controls (threefold increase in total cells and CD11c^+^ compared to the MSR with a lower‐aspect‐ratio). Moreover, the population of activated DC (CD11c^+^ and CD86^+^) in dLN increased about twofold. The serum levels of the OVA‐specific IgG1 and IgG2a also increased twofold, which confirmed the induction of the Th1 and Th2 adaptive immune responses. The vaccine enhanced the CTL population in the spleen threefold compared to the empty MSR. Finally, the C57BL/6 mice were immunized with the MSR vaccine and were inoculated 10 days later with EG7.OVA lymphoma cells in the back of the neck to study the material's tumor protective activity. The MSR vaccine inhibited fivefold tumor growth and prolonged animal survival (≈90% on day 30 after immunization) compared to the unencapsulated vaccine.

**Figure 6:**
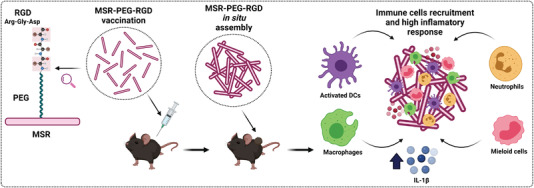
Surface‐modified MSR with PEG for enhancing the inflammatory response. PEG‐MSR auto‐assemble in vivo, induced secretion of cytokines and recruitment of immune cells. Adapted with permission.^[^ ^98^ ^]^ Copyright 2016, Elsevier.

1 year later, the same group studied the effect of the surface modification of MSR on modulating the immune response (**Figure** **6**).^[^ ^98^ ^]^ For that purpose, MSR were functionalized with 3‐aminoproyltriethoxysilane (APTES) to add primary amine groups, which were modified with methoxy‐terminated PEG12. The PEG‐modified MSR were then reacted with integrin‐binding ligand Arg‐Gly‐Asp (RGD), a peptide that mediates cell attachment. RGD/PEG modification induced more proinflammatory cytokine production (eightfold increase in interleukin‐1beta [IL‐1*β*]). In vivo studies revealed that PEG‐MSR scaffolds recruited more cells than the unmodified and PEG RGD/RDG modified scaffolds. However, the unmodified scaffolds contained more activated DC and macrophages (2.5‐fold increase in CD11c^+^ and CD86^+^) compared to the PEG‐modified MSR, which attracted a larger population of myeloid cells/neutrophils (1.3‐fold increase). This was attributed to the response of foreign material and of in vivo myeloid cells/neutrophils attraction to the scaffold. Overall, these results suggest that MSR surface chemistry determines the profile of immune cells infiltrated in the support and, thus, the vaccine's response.

**Figure 7:**
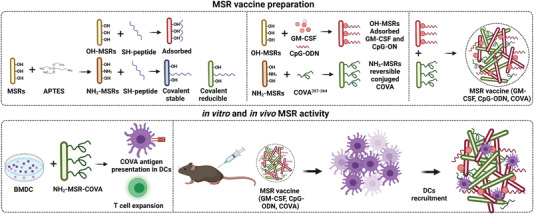
MSRs as an anticancer vaccination platform. Antigens are attached by covalent bonds to the surface of MSR and recruit DC to the injection area. Adapted with permission.^[^ ^99^ ^]^ Copyright 2018, ACS.

Based on modified MSR, Dellacherie et al. developed another cancer vaccine strategy based on DC‐recruiting MSR scaffolds modified with covalent‐conjugated peptides to enhance T‐cell responses against an antigen (**Figure** **7**).^[^ ^99^ ^]^ OVA‐derived cluster of differentiation 8 (CD8) and cluster of differentiation 4 (CD4) epitopes modified with cysteine (COVA275‐264) were covalently anchored to the MSR surface. The effect of the covalent conjugation of peptide on MSR was evaluated in vivo in a model in which CD1 mice were subcutaneously injected in the left flank. The results indicated that peptide retention had improved after covalent conjugation onto MSR compared to the adsorbed peptides and peptides alone (43%, 32%, and 2%, respectively). In a second step, the modified‐MSR scaffolds were loaded with GM‐CSF and CpG‐ODN. The recruitment of immune cells to the scaffold was evaluated in C57B16/J mice by a subcutaneous injection of the material in the right flank. A twofold increase in the OVA presenting DC (CD11b^+^ and CD11c^+^) occurred when the peptide was covalently conjugated compared to when it was adsorbed on MSR.

Following MSR surface modification, Li et al. used cationic polymer PEI as a strategy to enhance antigen immunogenicity.^[^ ^100^ ^]^ Functionalization with 60K PEI (MSRs‐PEI) improved the adjuvant properties of MSR, such as DC activation and cytokine production in vitro. CD86 and MHC‐II expression in BMDC increased ≈20%, while TNF‐*α* and IL‐1*β* production increased fourfold. Based on this improvement, these authors prepared cancer vaccines by separately adsorbing GM‐CSF, CpG‐ODN, and OVA on MSR and MSR‐PEI. The C57BL/6J mice were immunized with the vaccines to study the immunogenic effect of PEI in vivo. The results with MSR‐PEI showed enrichment in the DC cell population recruited in the dLN (twofold), circulating CTLs (twofold), and the ratio of effector T‐cells to Tregs (threefold). This scenario suggests that the MSR‐PEI vaccine improved host DC activation and antigen presentation by trafficking to secondary lymphoid organs and a cytotoxic effect against tumor cells. To evaluate the MSR‐PEI vaccine's antitumor immunity potential, it was loaded with a tumor‐specific antigen (a peptide that derived from the E7 oncoprotein of human papillomavirus) and was injected into C57BL/6 mice previously inoculated with the E7‐expressing TC‐1 carcinoma. The MSR‐PEI E7 vaccine caused complete tumor regression in most animals and about 80% survived. Immune memory efficacy and antimetastatic capacity were also confirmed. After 6 months, animals were inoculated with the same carcinoma cells and did not develop tumors. These results were also corroborated in lung metastasis models using tumor‐derived neoantigens, in which MSR‐PEI eradicated lung metastasis. Finally, vaccination with MSR‐PEI has enhanced anti‐CTLA‐4 immunotherapy when co‐administered. The overall results evidence the MSR‐PEI vaccine's potential to generate a strong cytotoxic effect against tumor cells.

More recently, Cheung et al. described a system that mimics natural APCs. It consists of a lipid bilayer (SLBs) supported by MSRs.^[^ ^101^ ^]^ MSRs were loaded with IL‐2, coated with lipid bilayers containing biotinylated lipids (MSRs‐SLBs) and mixed with streptavidin and biotinylated T‐cell activating cues (*α*CD3 and *α*CD28 for polyclonal T‐cell expansion, and peptide‐loaded MHC and *α*CD28 for antigen‐specific T‐cell expansion), obtaining a system that mimics natural APCs (APC‐ms). Compared to commercial CD3/CD28 T‐cell expansion beads (Dynabeads), APC‐ms led to the formation of considerable CD8‐biased T‐cell expansion (three‐ to five‐fold). Likewise, APC‐ms‐expanded T‐cells recognized their cognate antigen and killed both the B16‐F10 mouse melanoma cells and T2 human lymphoblast cells (80–100% cytotoxicity) in vitro. To evaluate the in vivo efficacy of the APC‐ms, human T‐cells expressing a CAR specific for CD19 (B‐cell specific antigen) (19BBz T‐cells) were restimulated with APC‐ms. Next NSG mice were inoculated with luciferase Raji cells to intravenously obtain a disseminated xenograft model of Burkitt's lymphoma. On day 4, mice were administered with 19BBz T‐cells restimulated with APC‐ms. IVIS imaging revealed on day 14 that no tumor‐derived bioluminescence was observed, which demonstrates the efficacy of restimulated CAR‐T 19BBz using the APC‐ms scaffold for reducing tumor cells. APC‐ms represents a potential adaptable platform for promoting more efficient cell expansion compared to conventional materials, such as dynabeads, and thus enhances cancer immunotherapy.

**Figure 8:**
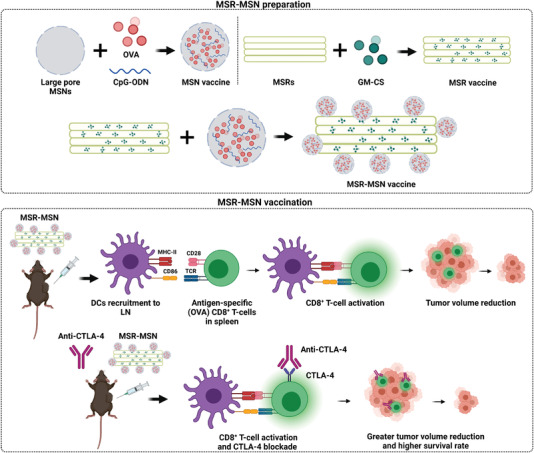
Illustration of a vaccine for cancer immunization formed by the MSR coupled to the MSN loaded with CpG ODN, OVA, and GM‐CS. The nanodevice recruits and activates the DC in lymph nodes, activates OVA‐specific CD8^+^ T‐cells, and reduces tumor volume. Adapted with permission.^[^ ^102^ ^]^ Copyright 2020, Elsevier.

Another strategy to improve cancer immunotherapy has been recently reported by Nguyen et al. It consists in combining MSR with MSN to put to the best use the benefits that both offer (**Figure** **8**).^[^ ^102^ ^]^ MSR were loaded with GM‐CS, whereas large‐pore MSNs were decorated with aminopropyl moieties and loaded with OVA and CpG‐ODN. Then both were mixed to yield the final vaccine (MSR‐MSN), where MSN were deposited onto the external surface of MSR. The MSR‐MSN vaccine induced the infiltration of DC on the scaffold (600 000 CD11c^+^ and CD11b^+^ cells in 7 days), the expression of both CD86 and MHC‐II (10% and 25% in CD11c^+^, respectively), and the increment in the CD11c^+^RITC^+^ cells in the inguinal lymph node (fivefold compared to the MSN vaccine in 1 day). The MSR‐MSN vaccine also exhibited significant tumor suppression (complete tumor ablation on day 15) and a higher survival rate (90% viability on day 26) in the C57BL/6 mice inoculated with B16‐OVA melanoma cells. The percentage of H‐2Kb OVA tetramer^+^ and IFN‐*γ* ^+^ among the CD8^+^ T‐cells in the spleen improved in the animals vaccinated with MSR‐MSN (twofold compared to the MSN vaccine). In addition, immunization with MSR‐MSN showed a synergy with the conventional anti‐CTLA‐4 therapy. Dual administration induced greater tumor volume reduction (1.5‐fold) and a higher survival rate (20% increment on day 25) than the separate administration. The results above dealing with the use of MSR‐based nanomaterials have proven that these promote efficient DC recruitment, T‐cell expansion, tumor removal, prevention from metastasis, and improvement in pre‐existing cancer treatments. These findings also show the very high potential of MRS as adaptable platforms to achieve the desired cancer‐vaccine responses only by making minor changes to them thanks to their very wide versatility.

### 3.2 Immune Cell Recruitment

Infiltration of immune cells in tumors can be promoted using chemokines, a type of cytokine that attracts and modulates specific subsets of effector leukocytes to develop antitumor immunity. Following this approach, two research groups have developed upconversion fluorescent nanoparticles (UCNPs) loaded with chemokine (C‐C motif) ligand 21 (CCL21) to induce the migration of immune cells to tumor cells.^[^ ^103^, ^104^ ^]^ UCNP were based on core–shell structured NaYF4 NPs co‐doped with lanthanide ions Yb/Er, modified with a uniform mesoporous silica coating. Additionally, the nanodevices were externally functionalized with amine groups, where folic acid (FA) was covalently conjugated (CCL21‐FA‐UCNPs). Lee et al. demonstrated that CCL21‐FA‐UCNP were able to cross an endothelial cell monolayer and specifically targeted an ovarian carcinoma cell line (OVCAR‐3) in an in vitro endothelial‐tumor cell bilayer model. They also found that nanodevices induced a 1.3‐fold increase in lymphocyte migration (Jurkat T‐cells) compared to the control. Years later, Wimalachandra et al. also confirmed the FA targeting effect in a breast cancer cell line (4T1) and evaluated NP diffusion across the endothelial barrier to selectively target OVCAR‐3 cells. These findings were corroborated in both 4T1 and OVCAR‐3 tumor‐bearing mice after the intravenous injection of nanodevices. Finally, the immune‐attractant ability of CCL21‐FA‐UCNP was studied in the microfluidic device. As expected, more Jurkat cells accumulated (≈60% increase compared to the control FA‐UCNP) and the DC expressing CCR7 (≈80% increase compared to the control FA‐UCNP) in the OVCAR‐3 cells compartment in response to CCL21‐FA‐UCNP. Taken together, these results indicate that employing nanomaterials to specifically deliver chemokines to tumors is a promising approach for immunotherapy due to their ability to host large molecules given the huge potential to induce immune cell trafficking across biological barriers and the TME.

### 3.3 Immunological Cell Death/Combinatorial Therapy

The final step in the cancer immunity cycle aims to kill cancer cells. In this way, MSN are potential tools to undergo different types of therapies to enhance antitumor therapy and, thus, maximize cancer cell death. Several approaches using MSN have been described by combining MSN‐based cancer vaccines with the delivery of conventional chemotherapeutics, and also with the inhibition of immune checkpoints by means of anti‐PD‐1 or anti‐PD‐L1 therapy. Recently, PDT‐ and PTT‐mediated immunotherapy have provided promising results in treating cancer considering their additional effect for stimulating antitumor immune responses as well as killing tumor cells.

#### 3.3.1 Chemo‐Immunotherapy

Zheng et al. evaluated the immunotherapeutic effect of a pH‐ and GSH‐activated nanosystem for the release of chemotherapeutic doxorubicin (DOX). These authors observed that this nanosystem triggered a stronger antitumor immune response than traditional chemotherapy.^[^ ^105^ ^]^ Similar results have been found by AbouAitah et al. when investigating the effects on the immune system of spherical‐shaped fibrous dendritic MSN (KCC‐1 type) loaded with chemotherapeutic colchicine and coated with a chitosan–glycine complex conjugated with FA.^[^ ^106^ ^]^ To enhance this effect of increased immune response to tumors using controlled drug delivery systems, NPs have been used as a synergistic platform to combine chemo‐immunotherapy by co‐loading chemotherapeutics and immunomodulators from the tumor environment. Kong et al. engineered biodegradable lipid‐coated HMSN (dHMSN) for the simultaneous release of DOX and the immunomodulators of tumor microenvironment transretinoic acid (ATRA) and IL‐2 (A/D/I‐dHMSN).^[^ ^107^ ^]^ In vivo studies of antitumor activity in C57BL/6 mice bearing B16F10 melanoma tumors have shown that tumor growth inhibition was 1.6‐fold enhanced in mice treated intravenously with A/D/I‐dHMLB compared to mice treated with DOX, ATRA, and IL‐2 unencapsulated. In another example, Lu et al. used MSN to develop a dual delivery system of oxaliplatin (OX, a chemotherapeutic drug) and indoximod (IND, an immunosuppressive IDO inhibitor), called OX/IND‐MSNP, to stimulate innate and adaptive immunity in pancreatic ductal adenocarcinoma.^[^ ^108^ ^]^ To evaluate its efficacy, orthotopically implanted KPC tumors in mice were treated with OX/IND‐MSNP. The results showed that the combination of OX/IND administration by MSN reduced primary tumor size eightfold by day 36 compared with free OX plus IND at equivalent doses.

**Figure 9:**
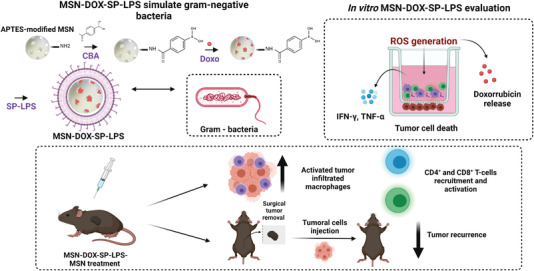
MSN‐DOX‐SP‐LPS as a chemo‐immunotherapy agent by generating ROS and DOX delivery. The nanodevice can induce the release of cytokines, activate tumor infiltrating macrophages and lymphocytes, and can inhibit tumor recurrence. Adapted with permission.^[^ ^109^ ^]^ Copyright 2017, Elsevier.

MSN have also served as a backbone for developing more innovative designs, Dong et al. engineered the first example of pathogen‐mimicking nanodevices to generate immune responses and reactive oxygen species (ROS) in the tumor microenvironment, and to achieve a stronger chemo‐immunotherapy effect (**Figure** **9**).^[^ ^109^ ^]^ MSR were functionalized with APTES and amine groups were then reacted with 4‐carboxyphenylboronic acid to develop ROS‐responsive drug‐release nanodevices. The pores of the functionalized MSN were loaded with DOX and conjugated with detoxified lipopolysaccharide (SP‐LPS) through a stable cyclic ester to simulate Gram‐negative bacterial infection to activate immune responses and to trigger ROS production (MSN‐DOX‐SP‐LPS). DOX release is due to ROS oxidizing the arylboronic ester. These authors evaluated the synergistic effect of chemotherapy and immunotherapy on H22 tumor‐bearing 5‐week‐old female Kunming mice. The animals treated intravenously with MSN‐DOX‐SP‐LPS showed threefold more effective tumor growth inhibition compared to the animals treated with the equivalent amount of free DOX or free SP‐LPS. The mechanism for anticancer activity could have been caused by the fact that the percentage of activated macrophages was significantly higher in the animals treated with MSN‐DOX‐SP‐LPS, which contributed to the recruitment and activation of CD4^+^ and CD8^+^ T‐cells, as demonstrated by an analysis of tumor‐infiltrating immune cells. The immune memory activity of the MSN‐DOX‐SP‐LPS NPs was also examined. For this purpose, a primary tumor model was established by the subcutaneous injection of H22 cells into the left flank of mice. After 7 days, mice were injected intravenously with MSN‐DOX‐SP‐LPS and the solid tumor was surgically removed 15 days later. In a final step, H22 cells were injected subcutaneously into the right flank of mice and tumor growth was monitored. The tumor recurrence percentage in the mice treated with MSN‐DOX‐SP‐LPS after 20 days was 2.5‐fold lower than in the group treated with PBS and MSN‐DOX NPs, which highlights the potential of the nanoconjugates to develop immune memory.

In a subsequent study, Xu et al. developed a revolutionary virus‐like mesoporous silica nanoparticles (VH‐MSNs), whose unique topological structure on the NP surface offered the advantage of enhancing cellular uptake and amplifying the immune response.^[^ ^110^ ^]^ VH‐MSN were synthesized using perovskites as self‐consuming templates in a bi‐phase reaction system. The process allowed a virus‐like morphology and topology to be obtained with three different structures: an inner hollow cavity, a shell, and 10‐nm nanotubes perpendicular to the shell. Then the VH‐MSN hollow cavity was loaded with DOX (DOX@VH‐MSNs). Cell viability assays performed in different cellular models indicated good biocompatibility for VH‐MSN and high cytotoxicity for DOX@VH‐MSN in a concentration‐dependent manner. Remarkably, the 4T1 breast tumor‐bearing mice treated with DOX@VH‐MSNs showed 3.5‐fold higher tumor growth inhibition than the mice treated with free DOX. Finally, the immunostimulatory effects of DOX@VH‐MSN were evaluated. It was concluded that treatment caused DC's maturation in axillary lymph nodes and improved cytokine secretion (IFN‐*γ* and interleukin‐6 [IL‐6]).

In conclusion, the use of mesoporous silica materials offers a very high potential for developing nanosystems based on chemo‐ and immuno‐therapy. Systems with large pore volumes allow chemotherapeutic and immunomodulatory co‐encapsulation, providing an excellent synergistic therapy with better outcomes than traditional chemotherapy. In addition, the surface of NPs can be chemically modified in many ways, which results in the generation of nanosystems like bacteria or viruses that enhance immunological effects.

#### 3.3.2 Checkpoint Blockade for ICD

Immunotherapy based on checkpoint blockade has been one of the most successful approaches to cancer treatment. The combination of MSN with these checkpoint inhibitors can have added value to effectively target and kill cancer cells. This concept was exploited for the first time by Choi et al.^[^ ^111^ ^]^ In this first approach, the anti‐PD‐L1 antibody was encapsulated using ultralarge pore MSN (UPMSNP). Pores were capped with ferumoxytol (Fer) (Fer‐ICB‐UPMSNP) to allow the sustained release of the drug and to provide magnetic resonance imaging (T2 contrast). The authors evaluated the synergistic antitumoral effect of the designed NPs in a Tramp C1 prostate cancer mice model by a sequential approach using standard chemotherapy, followed by treatment with Fer‐ICB‐UPMSNP NPs. After tumor formation, cabazitaxel (Cbz) chemotherapy was injected intratumorally into mice to increase the expression levels of immunogenic cell death markers and to achieve the up‐regulation of PD‐L1 in cancer cells. After 72 h, Fer‐ICB‐UPMSNPs were intratumorally injected and guided by magnetic resonance to the central tumor region. Tumor‐specific immune response was significantly activated with nanoimmunotherapy after Cbz treatment (5.5‐fold) compared to systemic anti‐PD‐L1 antibody administration (2.5‐fold) with subsequent greater tumor growth inhibition. These findings indicate the usefulness of using a high porous scaffold to encapsulate big immunotherapeutics, which allows drug protection to the target site and, thus, enhances the antitumoral effect.

Following a similar premise, using MSN with a large porous framework to protect and deliver immune checkpoint inhibitors, Li et al. developed metal–organic‐framework (MOF)‐gated MSN (MS@MOF) to generate durable antitumor immunity.^[^ ^112^ ^]^ This strategy combines the effect of cancer vaccines with systemic PD‐1 blockade therapy. For this purpose, the authors used a layer‐by‐layer self‐assembly route to integrate a cancer antigen (OVA) and an immunopotentiator (polyinosinic‐polycytidylic acid, polyIC) or a checkpoint inhibitor antibody (anti‐CTL4) into the stellated pore channels of a mesoporous silica core capped with a pH‐sensitive MOF gatekeeper composed of Zn^2+^ and 2‐methylimidazole. Different combinations of NPs were prepared to evaluate their antitumoral activity and immune response activation in a prophylactic mouse model. Although the NPs containing anti‐CTL4 achieved an excellent immunoresponse when compared with the negligible outcome of free anti‐CTL‐4, the cancer vaccine (MS@OVAinMOF)@(polyICinMOF) exhibited the best results. In a second step, the cancer nanovaccine was evaluated in a combined therapy with anti‐PD‐1 in an E.G7‐OVA tumor‐bearing mice model. The combined immunotherapeutic effect using NPs and a low anti‐PD‐1 dose (20 µg/mouse) was compared to free OVA plus anti‐PD‐1 at an equivalent dose (20 µg/mouse) or a higher dose (200 µg/mouse), respectively. The best results in tumor volume, survival rate (40 days compared to 20 days in the other groups), tumor volume and IL‐2 secretion (approximately eightfold‐change) in tumor, and CD8^+^ T‐cell population (approximately sixfold change) in spleen were exhibited by the mice treated with OVA and the high anti‐PD‐1 dose (200 µg/mouse), and interestingly with the nanovaccine plus anti‐PD‐1 at a lower dose. After studying specific cytotoxic CD8^+^ T‐lymphocyte activity (CTL) to E.G7.OVA‐cells, the data revealed that the mice injected with (MS@OVAinMOF)@(polyICinMOF) and anti‐PD‐1 presented the greatest cytotoxicity to E.G7‐OVA cancer cells.

**Figure 10:**
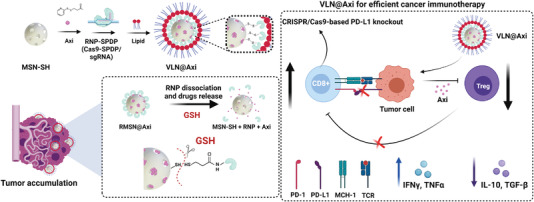
VLN‐sgPD‐L1@Axi for anticancer immunotherapy. Representation of preparation, delivery properties by the recognition of GSH, and suppression of Tregs to revert CD8^+^ T‐cells exhaustion. Adapted with permission.^[^ ^113^ ^]^ Copyright 2020, Elsevier.

Recently, virus‐like core–shell MSN were used by Li et al. to apply CRISPR‐Cas9‐based cancer immunotherapy (**Figure** **10**).^[^ ^113^ ^]^ MSN were modified with thiol groups and loaded with axitinib (Axi), an inhibitor of tyrosine kinase. The Cas9 protein was derivatized with succinimidyl‐3‐(2‐pyridyldithio) propionate and sgRNA that targets the PD‐L1 encoding gene (sgPD‐L1) and was conjugated to the surface of MSN through disulfide bonds. Finally, the system was coated with a lipid layer (made of DOTAP, DOPE and PEG2000‐DSPE) to protect the Cas9 system during blood circulation and to enhance cell internalization by tumor cells (VLN‐sgPD‐L1@Axi). The nanosystem's efficacy was evaluated in a tumor‐bearing mice model. C57BL/6 mice were subcutaneously inoculated with melanoma B16F10 cells in the mammary fat pad and intravenously injected with NPs. The VLN‐sgPD‐L1 nanosystem exhibited tumor growth suppression efficacy (<1000 mm^3^ compared to the control groups that obtained 2000 mm^3^). The Western blot and immunofluorescence analyses of tumor tissues confirmed the in vivo PD‐L1 knockout effect exhibited by VLN‐sgPD‐L1. Tumors had higher CD8+ infiltrating T‐lymphocytes and cytokine levels (IFN‐*γ* and TNF‐*α*) (≈2.5‐fold increase). Regarding the synergistic activity of VLN‐sgPD‐L1 combined with axitinib, the mice treated with VLN‐sgPD‐L1@Axi showed even higher survival rates and tumor growth suppression, with a reduction of 500 mm^3^ in tumor volume compared to the groups of monotherapies in which volumes were 1500 mm^3^. In addition to PD‐L1 editing, VLN‐sgPD‐L1@Axi down‐regulated the expression of axitinib targets pAKT and pERK, and the marker of suppressive Treg lymphocytes, VEGF, thus, resulting in the increase in infiltrated CD8^+^ T‐lymphocytes and cytokine secretion, and in a decrease in the immunosuppression associated with Tregs in the TME.

The few reports described above used MSN to deliver checkpoint blockade therapeutics, mainly in strategies combined with previous standard chemotherapy or vaccination. All these findings confirm the versatility of MSN as a scaffold to allow combining advanced strategies, such as immunotherapy via CRISPR‐Cas9 technology, as well as drug release for enhancing cancer treatment. MSN can also be suitable for previous ICD to enhance the efficacy of combined PD‐1 checkpoint blockade therapy. In this case, Xie et al. used MSN to perform starvation therapy, followed by PD‐1 checkpoint blockade for improved cancer therapy. MSN were loaded with glucose oxidase (GOx) (for starvation therapy) and coated with membranes from melanoma B16‐F10 cells (CMSN‐GOx).^[^ ^114^ ^]^ The combined treatment resulted in an improved survival rate in melanoma tumor‐bearing mice compared to the control groups, and also in significant decreased tumor growth. Besides, a significant increase in the CD80 and CD86 markers associated with DC maturation was found in the animals treated with CMSN‐GOx plus PD‐1 therapy (≈50% vs 30% in the PD‐1 therapy alone). These findings agree with the results obtained from the tumor‐infiltrating lymphocyte profile characterization and showed an effective CD4^+^ to CD8^+^ T‐cell ratio in the mice treated with combined therapy, approximately twofold compared to the PD‐1 monotherapy and, thus, confirms the stronger antitumor immune response.

#### 3.3.3 Photodynamic Immunotherapy

PDT is a noninvasive anticancer treatment that uses photosensitizers (PS) agents activated at a specific wavelength of light to generate cytotoxic ROS, which cause tumor cell death and release TAA, as well as DC recruitment. The main advantages of using nanotechnology in PDT lie in the increase of the PS at the target site, while reducing the toxicity of normal tissues/cells. In addition, controlled release allows a constant PS delivery rate to be maintained which, thus, enhances the effects of PDT.^[^ ^115^ ^]^

Considering the potential of PDT, advances in the integration of MSN with PS, such as merocyanine 540 (MC540) and chlorin e6 (Ce6), in combination with vaccine adjuvants has allowed the generation of promising nanovaccines to achieve PDT and immunological synergetic therapy. Im et al. engineered a hypoxia‐responsible nanodevice with Ce6 covalently attached in the inner pores of MSN and the CpG oligonucleotide adsorbed by electrostatic interactions (CAGE). Tumor growth inhibition was evaluated using B16.Mo5 cells subcutaneously inoculated in the right flank of C57BL/6 mice and NPs were intravenously administered.^[^ ^116^ ^]^ The group of mice treated with CAGE, plus laser irradiation, showed fourfold greater tumor growth inhibition than the mice treated with CAGE without light or CAGE (without CpG), plus laser, as well as a 100% survival rate for 28 days. In a similar study, Ding et al. designed a nanovaccine using large‐pore mesoporous‐silica‐coated *β*‐NaYF4:20%Yb,2%Er upconverting NPs (UCMS) co‐loaded with MC540 and CT26 TF as a tumor antigen of colorectal carcinoma (denoted as UCMSs‐MC450‐TF).^[^ ^117^ ^]^ To evaluate the potential immunoadjuvant of the nanovaccine, CT26tumor‐bearing Balb/c mice were subcutaneously immunized with three injections of UCMSs‐MC540‐TF, plus near‐infrared (NIR) laser irradiation. The studies revealed that, unlike the other control groups, the whole NP treatment achieved tumor clearance after 18 treatment days and a 100% survival rate after 65 days.

**Figure 11:**
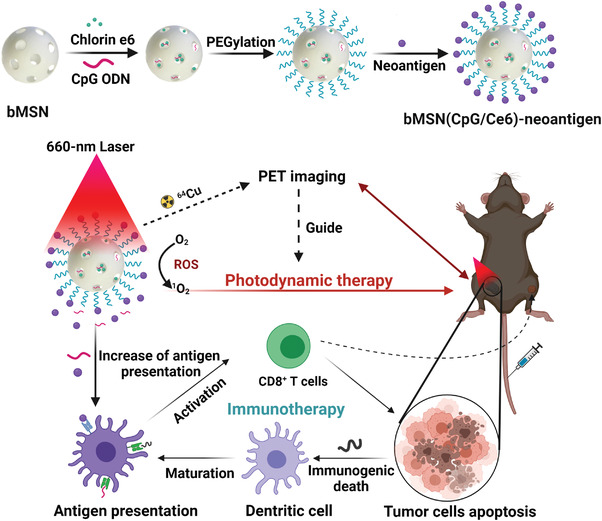
Theragnostic bMSN(CpG/Ce6)‐neoantigen nanovaccine for PET imaging and PDT cancer immunotherapy. After laser irradiation, the nanodevice can generate ROS by eradicating tumor cells by immunogenic cell death. Given the antigen presentation from nanoparticles, DC maturates and promotes lymphocytes T activation with subsequent tumor cancer cell death. Adapted with permission.^[^ ^118^ ^]^ Copyright 2019, ACS.

Furthermore, Xu and collaborators developed a nanovaccine for PDT‐immunotherapy combined with image‐guided therapy by positron emission tomography to facilitate quantitative cancer diagnosis (**Figure** **11**).^[^ ^118^ ^]^ Biodegradable MSNs (bMSNs) were synthesized using a heterogeneous oil–water biphase reaction system and the external surface was decorated with aminopropyl moieties. Mesopores were loaded with CpG and Ce6. The surface was modified with orthopyridyl disulfide PDP‐PEG‐succinimidyl ester, through the formation of amide bonds, and decorated with Adpgk (neoantigen peptide of the MC‐38 carcinoma tumor) by disulfide bond formation (bMSN(CpG/Ce6)‐Adpgk). Ce6‐loaded bMSN showed efficient singlet oxygen generation by 660 nm laser irradiation. The therapeutic efficacy of the bMSN vaccination was investigated using a bilateral two‐tumor model with MC‐38 colon cells. The C57BL/6 mice were inoculated with MC‐38 cells in the right flank, followed by the inoculation of MC‐38 cells in the contralateral flank on day 8. The first vaccination was administered intravenously via the tail vein on day 9, and only the tumor of the right flank was treated with laser irradiation. This was repeated on day 16. When laser irradiation combined with the bMSN(CpG/Ce6)‐Adpgk vaccine was used instead of a soluble vaccine (CpG, Ce6, and Adpgk free), tumor volume reduced ninefold. Moreover, median survival increased from 25 days to more than 40 days. Moreover, the greatest antigen‐specific CD8^+^ T‐cells response was observed in the PBMCs (peripheral blood mononuclear cells) from the animals treated with the bMSN vaccine (5.5‐fold greater than the soluble vaccine). Tumor‐infiltrating CD8^+^ T‐cells and activated intratumoral CD11^+^ CD86^+^ DC were detected at a high frequency (threefold higher than the soluble vaccine).

**Figure 12:**
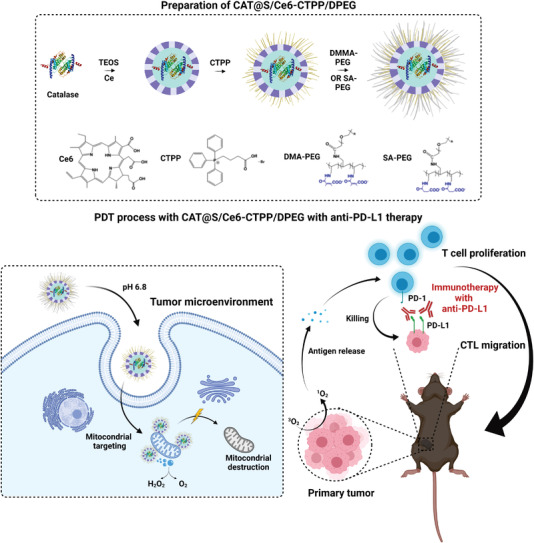
Scheme of CAT@S/Ce6‐CTPP/DPEG as a cancer PDT platform. The nanoreactor encapsuled an enzyme, a PS, and a mitochondrial targeting molecule, and is coated with a pH‐responsive polymer. In tumor cells, the nanoreactor is able to reach the mitochondria and generate ROS, which results in organelle destruction and hypoxia reversion. In addition, T‐cell proliferation is achieved when combined with anti‐PD‐L1 immunotherapy, antigen release, and CTL migration. Adapted with permission.^[^ ^119^ ^]^ Copyright 2018, ACS.

Another strategy consists of combining PS and checkpoint blockade immunotherapy using MSN. Following this concept, Yang et al. developed a smart nanoreactor system for PDT combined with antiPD‐L1 immunotherapy (**Figure** **12**).^[^ ^119^ ^]^ The system was based on hollow silica NPs with the enzyme catalase (CAT) encapsulated, as well as Ce6 doped into the silica network (CAT@S/Ce6). The mitochondrial targeting molecule (3‐carboxypropyl)triphenylphosphonium bromide, CTPP) was covalently conjugated to APTES, and then mixed with CAT@S/Ce6 to yield CAT@S/Ce6‐CTPP. Finally, CAT@S/Ce6‐CTPP was coated with the pH‐responsive anionic polymer, PEG/2,3‐dimethylmaleic anhydride‐*co*‐poly(allylamine hydrochloride) (DPEG), to give the final solid CAT@S/Ce6‐CTPP/DPEG. The authors demonstrated in NPs that CTPP allowed to target mitochondria, and CAT triggered the transformation of endogenous hydrogen peroxide (H_2_O_2_) into O_2_ to, thus, overcome hypoxia, which improved the effectiveness of PDT. Afterward, the synergistic effect of the combination of PDT using CAT@S/Ce6‐CTPP/DPEG and PD‐L1 checkpoint blockade immunotherapy was evaluated. For this purpose, Balb/c mice were subcutaneously injected with 4T1 cells into the left and right flanks of each mouse. After tumor formation, mice were treated with CAT@S/Ce6‐CTPP/DPEG, and the left‐flank tumors were irradiated with 660 nm light 24 h later. In addition, the anti‐PD‐L1 antibody was intravenously injected on days 1, 3, and 5. The results revealed that only the treatment with CAT@S/Ce6‐CTPP/DPEG, plus light, in combination with the antiPD‐L1 antibody generated fourfold more CTLs in the irradiated primary tumor and the non‐irradiated distant tumor (compared to the control treatments). Furthermore, only the synergistic treatment was able to suppress the growth of both tumors, which evidences the potential to inhibit metastasis.

The studies above have shown that using mesoporous silica materials for the co‐administration of PS and immunomodulators triggers a more effective antitumor response than separate administration. One main limitation of PDT is its use in disseminated cancer. However, the combination of PDT and immunotherapy in a nanodevice results in an effective synergistic effect to achieve complete primary tumor inhibition and the eradication of metastatic tumors.

#### 3.3.4 Chemo‐Dynamic Therapy

Following a similar concept, chemo‐dynamic therapy (CDT) has emerged as a potential strategy for killing cancer cells via the conversion of H_2_O_2_ into the harmful hydroxyl radical (a type of ROS) by Fenton/Fenton‐like reactions using CDT agents.^[^ ^120^ ^]^ In this way, Li et al. developed a nano‐catalytic system for the synergy between ferroptosis, an antitumor therapy that promotes TAA release, and immunotherapy.^[^ ^121^ ^]^ The authors used dendritic MSN (DMSN) loaded with ultrasmall CaO_2_ and Fe_3_O_4_ NPs, coated with a pH‐responsive lipid membrane (CaFe@DMSN/C). The coating consisted of a PEOz‐liposome prepared from lecithin, cholesterol, and 1,2‐distearoyl‐*sn*‐glycero‐3‐phosphoethanolamine (DSPE). In the acidic microenvironment of tumor tissues, the lipid coating was removed, followed by H_2_O_2_ production by CaO_2_ NPs, which was subsequently transformed by Fe_3_O_4_ NPs into harmful hydroxyl radicals through a Fenton‐like reaction to induce ferroptosis. Besides, the pH increment induced by proton consumption by NPs provoked the polarization of tumor‐associated macrophages from M2 (promoted tumor metastasis) to M1 (activated the antitumor immune response). To stimulate the efficiency of TAA uptake by phagocytes, NPs were also loaded with the anti‐CD47 antibody, an immune checkpoint blockade (CaFe/aCD47@DMSN/C). In vivo experiments were carried out in Balb/c mice subcutaneously injected with 4T1 tumor cells and intravenously injected with CaFe@DMSN/C. pH changes in tumor tissue revealed the ability of CaFe@DMSN/C to neutralize acidic the TME with subsequent M1 macrophage polarization. Furthermore, antitumor efficacy studies have demonstrated the superiority of the CaFe@DMSN/C system. While the mice treated with CaFe@DMSN/C were still alive on day 80, the mice in the other groups were almost all dead by 72 days. To evaluate the antimetastatic effect of CaFe/aCD47@DMSN/C NPs containing aCD47, 4T1 cells were intravenously injected. While no lung metastasis signs were observed in the CaFe/aCD47@DMSN/C treatment group, all the other groups displayed more than 30 metastatic nodules. Macrophage polarization and memory cells were increased 1.5‐ and 2‐fold, respectively, after the CaFe/aCD47@DMSN/C combination therapy (compared to the other groups). Complete tumor growth inhibition was achieved.

#### 3.3.5 Photothermal Immunotherapy

PTT, based on NIR light irradiation to induce cancer cell death, can also be combined to enhance the immune response in cancer therapy. MSN combining PTT with immunotherapeutics have been recently described by Ong et al. In this case, small gold NPs were adsorbed on the external surface of amino‐modified extralarge pore MSN (XL‐MSNs). Then thiolated CpG‐ODN, as a potent immunopotentiator, were loaded on Au@XL‐MSNs by gold‐thiol bonding. Finally, thiolated‐PEG was used to further decorate the surface of NPs to, thus, yield Au@XL‐MSN‐CpG/PEG.^[^ ^122^ ^]^ The immunostimulatory effect of Au@XL‐MSN‐CpG/PEG was evaluated in BMDC, and greater activation of DC was accomplished with increased levels of pro‐inflammatory cytokines. Finally, the therapeutic effect was evaluated in the B16‐F10 tumor‐bearing mice. After tumor formation, mice were treated with Au@XL‐MSN‐CpG/PEG and NIR irradiation was applied for 5 min. The results showed greater inhibition for tumor growth (≈700 mm^3^), as well as a longer survival time (until day 40) in mice compared to the control groups, where tumors were 1000–1500 mm^3^ and animals survived until day 20–25. Greater synergistic therapy efficacy was attributed mainly to the cytotoxic effect from PDT by destroying tumor cells, which released tumor antigens at tumor sites. This effect combined with the delivery of immunopotentiator CpG‐ODN also resulted in the greater activation of tumor‐residing DC.

In another innovative approach, Seth et al. prepared NIR‐responsive core–shell NPs for the photothermal induction of the TAA release from tumors and the delivery of immune adjuvant gardiquimod.^[^ ^123^ ^]^ The nanodevice was composed of polydopamine (PDA) core NPs (acting as a photothermal agent) coated with a mesoporous silica shell onto which gardiquimod was loaded and the external surface was coated with 1‐tetradecanol (Gardi‐mPDA). The nanomaterial capabilities were investigated in vivo in melanoma tumor‐bearing mice. Mice were intratumorally injected with PBS, PDA@SiO_2_, LT680‐mPDA, or Gardi‐mPDA and irradiated, or not, with an 808 nm laser (14 mW mm^−2^). The group treated with Gardi‐mPDA + NIR irradiation presented the highest survival rate (until day 45) and tumor growth completely inhibited compared to the controls with tumor volumes of ≈500 mm^3^ and a lower survival rate (until day 25). Moreover, when mice were later rechallenged on the opposite flank to generate a secondary tumor, more resistance to tumor growth was observed in the group injected with Gardi‐mPDA + NIR irradiation. These findings are mainly attributed to the ability of NPs in the triggered TAA release from tumor cells, as corroborated in the B16‐F10 cells treated with NPs in the presence of NIR. The results confirmed the ability of NPs to release the cargo, triggered by the melting of the 1‐tetradecanol shell, and to stimulate the tumor antigen release from the treated cells at the same time. Immunostimulatory capabilities were confirmed by BMDC activation after the treatment with the cell culture supernatant of B16‐F10, which contains TAAs as determined by increased IL‐6 production.

Similarly, Qian et al. designed biodegradable NPs for photothermal synergistic immunotherapy. For this purpose, the authors incorporated carbon nanodots (CDs) into mesoporous silica frameworks (CD@MSNs).^[^ ^124^ ^]^ Biodegradability studies of CD@MSNs showed gradual degradation, which accelerated after NIR‐laser irradiation (808 nm). The authors demonstrated increased tumor growth inhibition (<500 mm^3^) in 4T1 tumor‐bearing mice in the CD@MSNs + NIR‐treated mice (intravenous injection) and no lung metastasis after 14 treatment days compared to the control groups (tumor volumes ≈> 000 m^3^). The stronger immuno‐stimulatory effect was attributed to the fact that NIR promoted the biodegradation of NPs into small debris capable of absorbing large quantities of TAA to make organs immune and to promote immunotherapy compared to the typical MSN scaffold. The presence of CD45^+^ and CD49b^+^ cells (T‐cells) and activated NK cells (twofold increase) confirmed these findings. A remarkable increase in macrophages took place in the spleen, liver, and lungs.

Y. Zhang et al. developed a nanosystem for synergistic cancer immunotherapy by combining PTT and genotoxic chemotherapy.^[^ ^125^ ^]^ For this purpose, two NPs were prepared for primary and distant tumor treatment. One was based on the MSN loaded with indocyanine green (ICG, a photothermal agent for PTT) and sepantronium bromide (YM155, a surviving inhibitor that induces tumor cell death) to yield MSNs‐ICG‐YM155. For the second NP, the authors covalently anchored the anti‐CD47 antibody (which blocks CD47 expressed on cancer cells surfaces and promotes recognition of cancer cells by the immune system) by amide bonds to MSN containing magnetic NPs (MNP@nSiO_2_‐anti‐CD47). Both the immune responses and the antitumor effect induced by synergistic therapy were evaluated in vivo in B16F10 tumor‐bearing mice. For this purpose, a primary tumor model was established by a subcutaneous injection of B16F10 cells into the left flank region. A distant tumor model was developed 7 days later by injecting melanoma cells into the right flank. Significant tumor suppression (≈50% of tumor suppression) in both primary and distant tumors was observed after treatment with MSNs‐ICG‐YM155 + NIR + MNP@SiO_2_‐anti‐CD47, as was a prolonged survival time compared to the other groups and, remarkably, conjugation of anti‐CD47 in NPs resulted in ≈10% increased effectiveness compared with the free administration. The results can be ascribed as MSNs‐ICG‐YM155 + irradiation inducing the highest DC maturation level and higher levels of inflammatory cytokines and tumor‐infiltrating immune cells to, thus, enhance antitumor immune response.

The versatility of the MSN scaffold also allows the combination of different therapeutic approaches with imaging. Zhan and co‐workers designed core–shell copper sulfide (CuS) mesoporous silica nanocomposites loaded with perfluoropentane (PFP), CuS@mSiO_2_‐PFP‐PEG (CPPs), for photoacoustic, and ultrasound PTT and the imaging diagnosis of breast cancer.^[^ ^126^ ^]^ MDA‐MB‐231 tumor‐bearing mice were used in combination with immunotherapy agent anti‐PD‐1 (intraperitoneally injected daily). The photoacoustic and ultrasound signals generated by CPPs in tumor regions after laser irradiation confirmed that CPPs could be a very effective contrast agent for cancer diagnosis, while laser‐activated CPPs in mice produce substantial tumor cell apoptosis and low cell proliferation. Primary and distant tumors, indicators of metastasis, were suppressed with the combined therapy, which was not achieved by PTT alone. Survival rates (more than 50 days) improved. Furthermore, CPP‐based PTT + anti‐PD‐1 immunotherapy increased the percentage of cytotoxic T CD8^+^ cells in both cytokine secretion and DC maturation in primary and distant tumors. Overall, the antitumor effect of combined CPP‐based PTT and immunotherapy provoked a significant enhancement in all the antitumor parameters.

**Figure 13:**
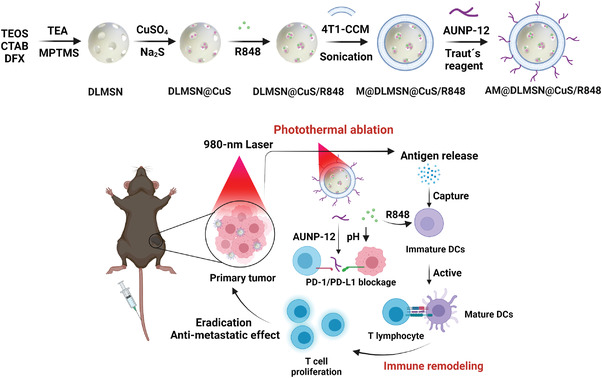
Illustration of synergic AM@DLMSN@CuS/R848 for photothermal therapy and immune remodeling in triple negative breast cancer. Laser irradiation induces immunogenic tumor cell death and the release of cancer antigens. This process provokes immune remodeling by promoting DC maturation, PD‐1/PD‐L1 blockage, and T‐cell proliferation with subsequent tumor eradication. Adapted with permission.^[^ ^127^ ^]^ Copyright 2020, ACS.

More recently, Cheng and collaborators designed a synergistic nanoplatform based on dendritic large‐pore MSN (DLMSN) to suppress triple‐negative breast cancer (TNBC) metastasis (**Figure** **13**).^[^ ^127^ ^]^ DLMSNs were loaded with CuS NPs, and the immune adjuvant resiquimod (R848). The loaded DLMSN were further coated with cancer cell membranes from mouse TNBC 4T1 cells and finally modified with antiPD‐1 peptide AUNP‐12. The final NPs (AM@DLMSN@CuS/R848) were capable of combining photothermal ablation and immune remodeling. The synergistic capabilities of AM@DLMSN@CuS/R848 were tested in vitro in co‐cultures of splenic lymphocytes and BMDC, 4T1 cells, and in a TNBC mice model with an excellent immune response and apoptosis in 4T1, which led to complete tumor eradication. In line with the research aim, to suppress TNBC metastasis, in a final assay a metastatic TNBC model was established by pretreatment with the subcutaneous injection of 4T1 cells into the right hips of female Balb/c mice and an intravenous injection of Luc‐4T1 cells to develop primary and lung metastatic tumors. The AM@DLMSN@CuS/R848 NIR‐treated mice manifested reduced growth for both primary and secondary tumors. Besides, the synergistic therapy triggered the best immune response, and the expressions of markers Ki67, CD8, and CD49 were found in the metastatic tumor, and blood (IFN‐*γ*, TNF‐*α*, and IL‐12p70) and CD44^+^CD62L^−^ memory T‐cells in the spleen. DC and T‐lymphocyte activation was confirmed in the TNBC mice model. Interestingly, the AM@DLMSN@CuS/R848‐irradiated group exhibited the fewest metastatic nodules, the greatest inhibition of Luc‐4T1 cells (75% cellular reduction), and enhanced infiltration of the CD8^+^ CLTs in the lung metastatic tumor. Taken together, these results reveal that the AM@DLMSN@CuS/R848‐based photothermal ablation of the primary tumor generates long‐term systemic antitumor immune responses, which can help to prevent metastases.

## 4 Conclusions and Outlook

Cancer immunotherapy emerges as an innovative precise treatment that is usually safer than traditional methods (surgery, chemotherapy, radiotherapy).^[^ ^28^ ^]^ Recent advances in immunotherapy have led to the FDA approving immunotherapy drugs, such as immune checkpoint inhibitors and CAR‐T, for a variety of cancer types.^[^ ^36^ ^]^ However, the drawbacks of the approved agents, such as patients' acquired resistance, toxicity, and low responses, highlight the need for new immunotherapy strategies. Nanotechnology has been an outbreak in this field and MSN have been demonstrated to be excellent platforms for the transportation and controlled release of large amounts of bioactive agents. Furthermore, mesoporous silica devices offer many advantages over other supports, such as enhanced biocompatibility, controllable biodegradability, improved targeting properties, and immune cell recruitment and activation.^[^ ^49^ ^]^

In this scenario, we herein reviewed the use of silica NPs in cancer immunotherapy applications and an increasing number of examples are expected in the near future. Overall, the results exhibit the potential of MSN with large porous scaffolds (HMSN or DMSN) to develop cancer vaccines, in which biomolecules like OVA, CpG‐ODN, or other TAA can be encapsulated to favor their protection and to enhance the immune response. Besides, MSN can serve as adjuvants to boost immunostimulation, which can also be enhanced by proper surface modification. In general, subcutaneous or intramuscular injections are used to administer cancer nanovaccines to obtain a better response because they promote a more efficient presentation of foreign bodies to macrophages and DC, and generate higher antibody titers.^[^ ^128^ ^]^ It is noteworthy that nanovaccines that target lymph nodes are a potential strategy to induce better immune responses. In this case, NPs from 10 to 80 nm, and modified with a neutral charge, improve targeting, and thus, cargo delivery to this system.^[^ ^129^ ^]^ In this way, intradermal administration, which favors the diffusion to lymph nodes, and the use of MSR have offered excellent results for developing a cancer vaccine.

Accordingly, at the beginning of cancer immunotherapy, initial nanoimmunotherapy focuses on the development of cancer vaccines, and extensive reports have been published. Moreover, in the last few years, and considering emerging novel therapies and the possibilities of mesoporous silica materials, advanced nanosystems have been developed. The unique characteristics of MSN allow the design of milliards of combined synergistic therapies to enhance not only immunotherapy, but also PDT, PTT, etc., for killing cancer cells. Despite as simple as possible nanodevices being preferred to clinical applications, complex systems exhibit remarkable effects and, in some cases, combined therapies results in the complete eradication of primary tumors and prevents metastasis.

Overall, these findings evidence the potential of MSN to be used to combine different synergistic therapies, and vaccine development with MSN as adjuvants or immune recruitment/modulating scaffolds, which may overcome many limitations of current approaches and create new therapeutic options for patients. Although increasing preclinical research with MSN has shed light on the safety and suitability of silica NPs for further clinical translation, there are still challenges to be overcome in clinical practice.^[^ ^44^, ^109^ ^]^ Until a few years ago, the scientific community assumed that MSN accumulated in the body with a poor removal rate. Nowadays, it is widely described that after MSN are administered, they usually tend to accumulate in major organs like liver, spleen, and lungs, and reach their target (tumor), regardless of the administration route, and are removed from the body by renal and hepatobiliary excretion.^[^ ^130^, ^131^ ^]^ All these recent findings suggest that MSN do not accumulate, which accomplishes FDA regulations to achieve clinics. In fact, in the last years, the number of clinical trials using silica NPs has remarkably increased.^[^ ^132^ ^]^ The advances made in developing bMSN by tuning their structure would be a key factor to take these NPs closer to real applications. Regarding synthetic procedures, another limiting factor to delay the clinical translation of MSN relies on scalability and reproducibility. Methods to carry out the reproducible manufacturing of MSN with narrow variability need to be established and industrial production has to be overcome.^[^ ^133^ ^]^

The first human trials with silica NPs have started a few years ago and most of them are still ongoing. Besides, NPs combined with immunotherapy are in the initial stage and more efforts are needed to elucidate the complexity in immune system‐nanomaterial interactions and the ability to intrinsically modulate innate and adaptive immunity, and to reinforce NPs' delivery to intended tissues.^[^ ^134^, ^135^ ^]^ The next few years will provide insight into the clinical potential of MSN in cancer nanoimmunotherapy to improve oncological patient care to develop highly efficacious personalized medicine.

## Conflict of Interest

The authors declare no conflict of interest.

## References

[1] S. Taefehshokr , A. Parhizkar , S. Hayati , M. Mousapour , A. Mahmoudpour , L. Eleid , D. Rahmanpour , S. Fattahi , H. Shabani , N. Taefehshokr , Pathol., Res. Pract. 2022, 229, 153723. 34952426 10.1016/j.prp.2021.153723

[2] M. Arruebo , N. Vilaboa , B. Sáez‐Gutierrez , J. Lambea , A. Tres , M. Valladares , Á. González‐Fernández , Cancers 2011, 3, 3279. 24212956 10.3390/cancers3033279 PMC3759197

[3] D. S. Chen , I. Mellman , Immunity 2013, 39, 1. 23890059 10.1016/j.immuni.2013.07.012

[4] M. M. Markiewski , R. A. DeAngelis , F. Benencia , S. K. Ricklin‐Lichtsteiner , A. Koutoulaki , C. Gerard , G. Coukos , J. D. Lambris , Nat. Immunol. 2008, 9, 1225. 18820683 10.1038/ni.1655 PMC2678913

[5] D. S. Chen , I. Mellman , Nature 2017, 541, 321. 28102259 10.1038/nature21349

[6] I. Adkins , J. Fucikova , A. D. Garg , P. Agostinis , R. Špíšek , Oncoimmunology 2015, 3, e968434. 25964865 10.4161/21624011.2014.968434 PMC4352954

[7] A. S. del Valle , A. Anel , J. Naval , I. Marzo , Front. Cell Dev. Biol. 2019, 7, 50. 31041312 10.3389/fcell.2019.00050 PMC6476910

[8] W. R. Heath , F. R. Carbone , Nat. Rev. Immunol. 2001, 1, 126. 11905820 10.1038/35100512

[9] A. Garcia‐Lora , I. Algarra , F. Garrido , J. Cell. Physiol. 2003, 195, 346. 12704644 10.1002/jcp.10290

[10] B. Seliger , M. J. Maeurer , S. Ferrone , Immunol. Today 2000, 21, 455. 10953098 10.1016/s0167-5699(00)01692-3

[11] M. J. Butte , M. E. Keir , T. B. Phamduy , A. H. Sharpe , G. J. Freeman , Immunity 2007, 27, 111. 17629517 10.1016/j.immuni.2007.05.016 PMC2707944

[12] K. A. Cycon , J. L. Clements , R. Holtz , H. Fuji , S. P. Murphy , Immunology 2009, 128, e641. 19740325 10.1111/j.1365-2567.2009.03052.x PMC2753899

[13] L. Galluzzi , E. Vacchelli , J.‐M. B.‐S. Pedro , A. Buqué , L. Senovilla , E. E. Baracco , N. Bloy , F. Castoldi , J.‐P. Abastado , P. Agostinis , R. N. Apte , F. Aranda , M. Ayyoub , P. Beckhove , J.‐Y. Blay , L. Bracci , A. Caignard , C. Castelli , F. Cavallo , E. Celis , V. Cerundolo , A. Clayton , M. P. Colombo , L. Coussens , M. V. Dhodapkar , A. M. Eggermont , D. T. Fearon , W. H. Fridman , J. Fučíková , D. I. Gabrilovich , et al., Oncotarget 2014, 5, 12472.25537519

[14] R. F. Rousseau , C. Hirschmann‐Jax , S. Takahashi , M. K. Brenner , Hematol./Oncol. Clin. North Am. 2001, 15, 741. 10.1016/s0889-8588(05)70245-8 PMC6361626 11676282

[15] FDA , BCG LIVE (For Intravesical Use), https://www.fda.gov/downloads/biologicsbloodvaccines/vaccines/approvedproducts/ucm163039.pdf.

[16] FDA , PROVENGE: Highlights of Prescribing Information, https://www.fda.gov/downloads/biologicsbloodvaccines/cellulargenetherapyproducts/approvedproducts/UCM210031.pdf, (accessed: April 2022).

[17] FDA , IMLYGIC: Highlights of Prescribing Information, https://www.fda.gov/downloads/BiologicsBloodVaccines/CellularGeneTherapyProducts/ApprovedProducts/UCM469575.pdf, (accessed: April 2022).

[18] B. Farhood , M. Najafi , K. Mortezaee , J. Cell. Physiol. 2019, 234, 8509. 30520029 10.1002/jcp.27782

[19] B. Rowshanravan , N. Halliday , D. M. Sansom , Blood 2018, 131, 58. 29118008 10.1182/blood-2017-06-741033 PMC6317697

[20] M. F. Sanmamed , F. Pastor , A. Rodriguez , J. L. Perez‐Gracia , M. E. Rodriguez‐Ruiz , M. Jure‐Kunkel , I. Melero , Semin. Oncol. 2015, 42, 640. 26320067 10.1053/j.seminoncol.2015.05.014

[21] S. A. Rosenberg , J. Immunol. 2014, 192, 5451. 24907378 10.4049/jimmunol.1490019 PMC6293462

[22] C. Y. Slaney , M. H. Kershaw , P. K. Darcy , Cancer Res. 2014, 74, 7168. 25477332 10.1158/0008-5472.CAN-14-2458

[23] Y. Huang , S. Goel , D. G. Duda , D. Fukumura , R. K. Jain , Cancer Res. 2013, 73, 2943. 23440426 10.1158/0008-5472.CAN-12-4354 PMC3655127

[24] K. L. Meadows , H. I. Hurwitz , Cold Spring Harbor Perspect. Med. 2012, 2, a006577. 10.1101/cshperspect.a006577 PMC3475399 23028128

[25] J. D. Martin , H. Cabral , T. Stylianopoulos , R. K. Jain , Nat. Rev. Clin. Oncol. 2020, 17, 251. 32034288 10.1038/s41571-019-0308-z PMC8272676

[26] C. Y. Slaney , P. Wang , P. K. Darcy , M. H. Kershaw , Cancer Discovery 2018, 8, 924. 30012854 10.1158/2159-8290.CD-18-0297

[27] R. S. Riley , C. H. June , R. Langer , M. J. Mitchell , Nat. Rev. Drug Discovery 2019, 18, 175. 30622344 10.1038/s41573-018-0006-z PMC6410566

[28] C. Pan , H. Liu , E. Robins , W. Song , D. Liu , Z. Li , L. Zheng , J. Hematol. Oncol. 2020, 13, 29. 32245497 10.1186/s13045-020-00862-w PMC7119170

[29] Z. J. Roberts , M. Better , A. Bot , M. R. Roberts , A. Ribas , Leuk. Lymphoma 2018, 59, 1785. 29058502 10.1080/10428194.2017.1387905

[30] P. Abdou , Z. Wang , Q. Chen , A. Chan , D. R. Zhou , V. Gunadhi , Z. Gu , Wiley Interdiscip. Rev.: Nanomed. Nanobiotechnol. 2020, 12, e1632. 32255276 10.1002/wnan.1632 PMC7725287

[31] A. D. Waldman , J. M. Fritz , M. J. Lenardo , Nat. Rev. Immunol. 2020, 20, 651. 32433532 10.1038/s41577-020-0306-5 PMC7238960

[32] F. Finkelmeier , O. Waidmann , J. Trojan , Expert Rev. Anticancer Ther. 2018, 18, 1169. 30304963 10.1080/14737140.2018.1535315

[33] L. Khoja , M. O. Butler , S. P. Kang , S. Ebbinghaus , A. M. Joshua , J. Immunother. Cancer 2015, 3, 36. 26288737 10.1186/s40425-015-0078-9 PMC4539882

[34] M. A. Postow , J. Chesney , A. C. Pavlick , C. Robert , K. Grossmann , D. McDermott , G. P. Linette , N. Meyer , J. K. Giguere , S. S. Agarwala , M. Shaheen , M. S. Ernstoff , D. Minor , A. K. Salama , M. Taylor , P. A. Ott , L. M. Rollin , C. Horak , P. Gagnier , J. D. Wolchok , F. S. Hodi , N. Engl. J. Med. 2015, 372, 2006. 25891304 10.1056/NEJMoa1414428 PMC5744258

[35] B. S. Guerrouahen , C. Maccalli , C. Cugno , S. Rutella , E. T. Akporiaye , Front. Oncol. 2020, 9, 1554. 32039024 10.3389/fonc.2019.01554 PMC6985581

[36] M. Liu , F. Guo , Precis. Clin. Med. 2018, 1, 65. 30687562 10.1093/pcmedi/pby011 PMC6333045

[37] K. P. Papadopoulos , F. Y.‐C. Tsai , T. M. Bauer , L. Muigai , Y. Liang , M. K. Bennett , K. W. Orford , S. Fu , J. Clin. Oncol. 2017, 35, 3005.

[38] S. M. Steggerda , M. K. Bennett , J. Chen , E. Emberley , T. Huang , J. R. Janes , W. Li , A. L. MacKinnon , A. Makkouk , G. Marguier , P. J. Murray , S. Neou , A. Pan , F. Parlati , M. L. M. Rodriguez , L.‐A. Van de Velde , T. Wang , M. Works , J. Zhang , W. Zhang , M. I. Gross , J. Immunother. Cancer 2017, 5, 101. 29254508 10.1186/s40425-017-0308-4 PMC5735564

[39] R. K. Kelley , E. Gane , E. Assenat , J. Siebler , P. R. Galle , P. Merle , I. O. Hourmand , A. Cleverly , Y. Zhao , I. Gueorguieva , M. Lahn , S. Faivre , K. A. Benhadji , G. Giannelli , Clin. Transl. Gastroenterol. 2019, 10, e00056. 31295152 10.14309/ctg.0000000000000056 PMC6708671

[40] S. Herbertz , J. S. Sawyer , A. J. Stauber , I. Gueorguieva , K. E. Driscoll , S. T. Estrem , A. L. Cleverly , D. Desaiah , S. C. Guba , K. A. Benhadji , C. A. Slapak , M. M. Lahn , Drug Des., Dev. Ther. 2015, 9, 4479. 10.2147/DDDT.S86621 PMC4539082 26309397

[41] L. Milling , Y. Zhang , D. J. Irvine , Adv. Drug Delivery Rev. 2017, 114, 79. 10.1016/j.addr.2017.05.011 PMC5647831 28545888

[42] S. Wang , Z. Sun , Y. Hou , Adv. Healthcare Mater. 2021, 10, 2000845. 10.1002/adhm.202000845 32790039

[43] B. De Angelis , N. Depalo , F. Petronella , C. Quintarelli , M. L. Curri , R. Pani , A. Calogero , F. Locatelli , L. De Sio , J. Mater. Chem. B 2020, 8, 1823. 32067013 10.1039/c9tb02246e

[44] Z. Zhao , L. Zheng , W. Chen , W. Weng , J. Song , J. Ji , J. Hematol. Oncol. 2019, 12, 126. 31779642 10.1186/s13045-019-0817-3 PMC6883629

[45] Z. Wen , F. Liu , Q. Chen , Y. Xu , H. Li , S. Sun , Biomater. Sci. 2019, 7, 4414. 31364635 10.1039/c9bm00961b

[46] Z. Gu , C. G. Da Silva , K. Van der Maaden , F. Ossendorp , L. J. Cruz , Pharmaceutics 2020, 12, 1054. 10.3390/pharmaceutics12111054 PMC7694212 33158166

[47] A. Karabasz , M. Bzowska , K. Szczepanowicz , Int. J. Nanomed. 2020, 15, 8673. 10.2147/IJN.S231477 PMC7654520 33192061

[48] Y. Liu , B. M. Crawford , T. Vo‐Dinh , Immunotherapy 2018, 10, 1175. 30236026 10.2217/imt-2018-0029

[49] T. L. Nguyen , Y. Choi , J. Kim , Adv. Mater. 2019, 31, 1803953. 10.1002/adma.201803953 30417454

[50] L. Wang , J. Liu , Biomater. Sci. 2021, 9, 1104. 33201163 10.1039/d0bm01676d

[51] Y. H. Chung , H. Cai , N. F. Steinmetz , Adv. Drug Delivery Rev. 2020, 156, 214. 10.1016/j.addr.2020.06.024 PMC7320870 32603813

[52] D. J. Irvine , E. L. Dane , Nat. Rev. Immunol. 2020, 20, 321. 32005979 10.1038/s41577-019-0269-6 PMC7536618

[53] W. Fan , B. Yung , P. Huang , X. Chen , Chem. Rev. 2017, 117, 13566. 29048884 10.1021/acs.chemrev.7b00258

[54] N. Bertrand , J. Wu , X. Xu , N. Kamaly , O. C. Farokhzad , Adv. Drug Delivery Rev. 2014, 66, 2. 10.1016/j.addr.2013.11.009 PMC4219254 24270007

[55] A. K. Iyer , G. Khaled , J. Fang , H. Maeda , Drug Discovery Today 2006, 11, 812. 16935749 10.1016/j.drudis.2006.07.005

[56] J. J. Moon , B. Huang , D. J. Irvine , Adv. Mater. 2012, 24, 3724. 22641380 10.1002/adma.201200446 PMC3786137

[57] Y. Li , C. Ayala‐Orozco , P. R. Rauta , S. Krishnan , Nanoscale 2019, 11, 17157. 31531445 10.1039/c9nr05371a PMC6778734

[58] H. Wang , D. J. Mooney , Nat. Mater. 2018, 17, 761. 30104668 10.1038/s41563-018-0147-9

[59] V. Manolova , A. Flace , M. Bauer , K. Schwarz , P. Saudan , M. F. Bachmann , Eur. J. Immunol. 2008, 38, 1404. 18389478 10.1002/eji.200737984

[60] Z. Amoozgar , M. S. Goldberg , Adv. Drug Delivery Rev. 2015, 91, 38. 10.1016/j.addr.2014.09.007 25280471

[61] Y. Zhuang , Y. Ma , C. Wang , L. Hai , C. Yan , Y. Zhang , F. Liu , L. Cai , J. Controlled Release 2012, 159, 135. 10.1016/j.jconrel.2011.12.017 22226776

[62] Y. Chen , H. Chen , J. Shi , Adv. Mater. 2013, 25, 3144. 23681931 10.1002/adma.201205292

[63] A. García‐Fernández , E. Aznar , R. Martínez‐Máñez , F. Sancenón , Small 2020, 16, 1902242. 10.1002/smll.201902242 31846230

[64] A. Bernardos , E. Piacenza , F. Sancenón , M. Hamidi , A. Maleki , R. J. Turner , R. Martínez‐Máñez , Small 2019, 15, 1900669. 10.1002/smll.201900669 31033214

[65] L. Pla , B. Lozano‐Torres , R. Martínez‐Máñez , F. Sancenón , J. V. Ros‐Lis , Sensors 2019, 19, 5138. 10.3390/s19235138 PMC6929179 31771224

[66] À. Ribes , E. Aznar , S. Santiago‐Felipe , E. Xifre‐Perez , M. Á. Tormo‐Mas , J. Pemán , L. F. Marsal , R. Martínez‐Máñez , ACS Sens. 2019, 4, 1291. 31020831 10.1021/acssensors.9b00169

[67] B. de Luis , A. Llopis‐Lorente , F. Sancenón , R. Martínez‐Máñez , Chem. Soc. Rev. 2021, 50, 8829. 34109333 10.1039/d0cs01048k

[68] B. de Luis , A. Llopis‐Lorente , P. Rincón , J. Gadea , F. Sancenón , E. Aznar , R. Villalonga , J. R. Murguía , R. Martínez‐Máñez , Angew. Chem., Int. Ed. 2019, 58, 14986. 10.1002/anie.201908867 31424153

[69] B. de Luis , Á. Morellá‐Aucejo , A. Llopis‐Lorente , T. M. Godoy‐Reyes , R. Villalonga , E. Aznar , F. Sancenón , R. Martínez‐Máñez , Chem. Sci. 2021, 12, 1551. 10.1039/d0sc04743k PMC8179104 34163918

[70] B. de Luis , Á. Morellá‐Aucejo , A. Llopis‐Lorente , J. Martínez‐Latorre , F. Sancenón , C. López , J. R. Murguía , R. Martínez‐Máñez , Nano Lett. 2022, 22, 1836. 35171622 10.1021/acs.nanolett.1c02435 PMC9940291

[71] Z. Li , Y. Zhang , N. Feng , Expert Opin. Drug Delivery 2019, 16, 219. 10.1080/17425247.2019.1575806 30686075

[72] C. Chircov , A. Spoială , C. Păun , L. Crăciun , D. Ficai , A. Ficai , E. Andronescu , Ş. C. Turculeƫ , Molecules 2020, 25, 3814. 10.3390/molecules25173814 PMC7503268 32825791

[73] E. Aznar , M. Oroval , L. Pascual , J. R. Murguía , R. Martínez‐Máñez , F. Sancenón , Chem. Rev. 2016, 116, 561. 26730615 10.1021/acs.chemrev.5b00456

[74] R. R. Castillo , D. Lozano , B. González , M. Manzano , I. Izquierdo‐Barba , M. Vallet‐Regí , Expert Opin. Drug Delivery 2019, 16, 415. 10.1080/17425247.2019.1598375 PMC6667337 30897978

[75] A. Llopis‐Lorente , B. Lozano‐Torres , A. Bernardos , R. Martínez‐Máñez , F. Sancenón , J. Mater. Chem. B 2017, 5, 3069. 32263705 10.1039/c7tb00348j

[76] P. Jain , N. Hassan , Z. Iqbal , F. Dilnawaz , Recent Pat. Drug Delivery Formulation 2018, 12, 228. 10.2174/1872211313666181203152859 30501606

[77] R. R. Castillo , M. Vallet‐Regí , Biotechnol. J. 2021, 16, 1900438. 10.1002/biot.201900438 33079451

[78] X. Wang , X. Li , A. Ito , Y. Watanabe , Y. Sogo , N. M. Tsuji , T. Ohno , Angew. Chem., Int. Ed. Engl. 2016, 55, 1899. 26404897 10.1002/anie.201506179

[79] G. Navarro‐Tovar , G. Palestino , S. Rosales‐Mendoza , Expert Rev. Vaccines 2016, 15, 1449. 27160927 10.1080/14760584.2016.1188009

[80] K. T. Mody , A. Popat , D. Mahony , A. S. Cavallaro , C. Yu , N. Mitter , Nanoscale 2013, 5, 5167. 23657437 10.1039/c3nr00357d

[81] Y. Xu , P. Claiden , Y. Zhu , H. Morita , N. Hanagata , Sci. Technol. Adv. Mater. 2015, 16, 045006. 27877826 10.1088/1468-6996/16/4/045006 PMC5090185

[82] H. Zhang , T. Cheng , L. Lai , S. Deng , R. Yu , L. Qiu , J. Zhou , G. Lu , C. Zhi , J. Chen , Nanoscale 2018, 10, 14516. 30024003 10.1039/c8nr03820a

[83] H. Zheng , S. Wen , Y. Zhang , Z. Sun , PLoS One 2015, 10, e0140265. 26451735 10.1371/journal.pone.0140265 PMC4599948

[84] X. Wang , X. Li , K. Yoshiyuki , Y. Watanabe , Y. Sogo , T. Ohno , N. M. Tsuji , A. Ito , Adv. Healthcare Mater. 2016, 5, 1169. 10.1002/adhm.201670051 27226038

[85] X. Zhou , Q. Su , H. Zhao , X. Cao , Y. Yang , W. Xue , Mol. Pharmaceutics 2020, 17, 4603. 10.1021/acs.molpharmaceut.0c00802 33175556

[86] X. Li , X. Wang , Y. Sogo , T. Ohno , K. Onuma , A. Ito , Adv. Healthcare Mater. 2013, 2, 863. 10.1002/adhm.201200149 23296515

[87] Y. Yang , M. Jambhrunkar , P. L. Abbaraju , M. Yu , M. Zhang , C. Yu , Adv. Healthcare Mater. 2017, 6, 1700466. 10.1002/adhm.201700466 28557331

[88] X. Wang , X. Li , A. Ito , K. Yoshiyuki , Y. Sogo , Y. Watanabe , A. Yamazaki , T. Ohno , N. M. Tsuji , Small 2016, 12, 3510. 27191183 10.1002/smll.201600677

[89] Q. Liu , Y. Zhou , M. Li , L. Zhao , J. Ren , D. Li , Z. Tan , K. Wang , H. Li , M. Hussain , L. Zhang , G. Shen , J. Zhu , J. Tao , ACS Appl. Mater. Interfaces 2019, 11, 47798. 31773941 10.1021/acsami.9b19446

[90] J. Xie , C. Yang , Q. Liu , J. Li , R. Liang , C. Shen , Y. Zhang , K. Wang , L. Liu , K. Shezad , M. Sullivan , Y. Xu , G. Shen , J. Tao , J. Zhu , Z. Zhang , Small 2017, 13, 1701741. 10.1002/smll.201701741 28861951

[91] P. L. Abbaraju , A. K. Meka , H. Song , Y. Yang , M. Jambhrunkar , J. Zhang , C. Xu , M. Yu , C. Yu , J. Am. Chem. Soc. 2017, 139, 6321. 28440642 10.1021/jacs.6b12622

[92] J. Y. Lee , M. K. Kim , T. L. Nguyen , J. Kim , ACS Appl. Mater. Interfaces 2020, 12, 34658. 32662625 10.1021/acsami.0c09484

[93] Y. Yang , Y. Lu , P. L. Abbaraju , J. Zhang , M. Zhang , G. Xiang , C. Yu , Angew. Chem., Int. Ed. Engl. 2017, 56, 8446. 28467690 10.1002/anie.201701550

[94] Y. Lu , Y. Yang , Z. Gu , J. Zhang , H. Song , G. Xiang , C. Yu , Biomaterials 2018, 175, 82. 29803106 10.1016/j.biomaterials.2018.05.025

[95] X. Wang , X. Li , A. Ito , Y. Sogo , Y. Watanabe , N. M. Tsuji , T. Ohno , ACS Appl. Mater. Interfaces 2017, 9, 43538. 29192493 10.1021/acsami.7b16118

[96] X. Wang , X. Li , A. Ito , Y. Sogo , Y. Watanabe , K. Hashimoto , A. Yamazaki , T. Ohno , N. M. Tsuji , Chem. Commun. 2018, 54, 1057. 10.1039/c7cc08222c 29323387

[97] J. Kim , W. A. Li , Y. Choi , S. A. Lewin , C. S. Verbeke , G. Dranoff , D. J. Mooney , Nat. Biotechnol. 2015, 33, 64. 25485616 10.1038/nbt.3071 PMC4318563

[98] W. A. Li , B. Y. Lu , L. Gu , Y. Choi , J. Kim , D. J. Mooney , Biomaterials 2016, 83, 249. 26784009 10.1016/j.biomaterials.2016.01.026 PMC4754159

[99] M. O. Dellacherie , A. W. Li , B. Y. Lu , D. J. Mooney , Bioconjugate Chem. 2018, 29, 733. 10.1021/acs.bioconjchem.7b00656 29318872

[100] A. W. Li , M. C. Sobral , S. Badrinath , Y. Choi , A. Graveline , A. G. Stafford , J. C. Weaver , M. O. Dellacherie , T.‐Y. Shih , O. A. Ali , J. Kim , K. W. Wucherpfennig , D. J. Mooney , Nat. Mater. 2018, 17, 528. 29507416 10.1038/s41563-018-0028-2 PMC5970019

[101] A. S. Cheung , D. K. Y. Zhang , S. T. Koshy , D. J. Mooney , Nat. Biotechnol. 2018, 36, 160. 29334370 10.1038/nbt.4047 PMC5801009

[102] T. L. Nguyen , B. G. Cha , Y. Choi , J. Im , J. Kim , Biomaterials 2020, 239, 119859. 32070828 10.1016/j.biomaterials.2020.119859

[103] K. Y. Lee , E. Seow , Y. Zhang , Y. C. Lim , Biomaterials 2013, 34, 4860. 23562047 10.1016/j.biomaterials.2013.03.029

[104] D. C. Wimalachandra , Y. Li , J. Liu , S. Shikha , J. Zhang , Y.‐C. Lim , Y. Zhang , ACS Appl. Mater. Interfaces 2019, 11, 37513. 31547654 10.1021/acsami.9b15178

[105] D.‐W. Zheng , J.‐L. Chen , J.‐Y. Zhu , L. Rong , B. Li , Q. Lei , J.‐X. Fan , M.‐Z. Zou , C. Li , S.‐X. Cheng , Z. Xu , X.‐Z. Zhang , Nano Lett. 2016, 16, 4341. 27327876 10.1021/acs.nanolett.6b01432

[106] K. AbouAitah , H. A. Hassan , A. Swiderska‐Sroda , L. Gohar , O. G. Shaker , J. Wojnarowicz , A. Opalinska , J. Smalc‐Koziorowska , S. Gierlotka , W. Lojkowski , Cancers 2020, 12, 144. 10.3390/cancers12010144 PMC7017376 31936103

[107] M. Kong , J. Tang , Q. Qiao , T. Wu , Y. Qi , S. Tan , X. Gao , Z. Zhang , Theranostics 2017, 7, 3276. 28900509 10.7150/thno.19987 PMC5595131

[108] J. Lu , X. Liu , Y.‐P. Liao , F. Salazar , B. Sun , W. Jiang , C. H. Chang , J. Jiang , X. Wang , A. M. Wu , H. Meng , A. E. Nel , Nat. Commun. 2017, 8, 1811. 29180759 10.1038/s41467-017-01651-9 PMC5703845

[109] K. Dong , Z. Li , H. Sun , E. Ju , J. Ren , X. Qu , Mater. Today 2017, 20, 346.

[110] D. Xu , X. Song , J. Zhou , X. Ouyang , J. Li , D. Deng , Colloids Surf., B 2021, 197, 111452. 10.1016/j.colsurfb.2020.111452 33189035

[111] B. Choi , H. Jung , B. Yu , H. Choi , J. Lee , D.‐H. Kim , Small 2019, 15, 1904378. 10.1002/smll.201904378 PMC7027959 31697036

[112] X. Li , X. Wang , A. Ito , N. M. Tsuji , Nat. Commun. 2020, 11, 3858. 32737343 10.1038/s41467-020-17637-z PMC7395732

[113] Q. Liu , C. Wang , Y. Zheng , Y. Zhao , Y. Wang , J. Hao , X. Zhao , K. Yi , L. Shi , C. Kang , Y. Liu , Biomaterials 2020, 258, 120275. 32798741 10.1016/j.biomaterials.2020.120275

[114] W. Xie , W.‐W. Deng , M. Zan , L. Rao , G.‐T. Yu , D.‐M. Zhu , W.‐T. Wu , B. Chen , L.‐W. Ji , L. Chen , K. Liu , S.‐S. Guo , H.‐M. Huang , W.‐F. Zhang , X. Zhao , Y. Yuan , W. Dong , Z.‐J. Sun , W. Liu , ACS Nano 2019, 13, 2849. 30803232 10.1021/acsnano.8b03788

[115] H. Abrahamse , C. A. Kruger , S. Kadanyo , A. Mishra , Photomed. Laser Surg. 2017, 35, 581. 28937916 10.1089/pho.2017.4308

[116] S. Im , J. Lee , D. Park , A. Park , Y.‐M. Kim , W. J. Kim , ACS Nano 2019, 13, 476. 30563320 10.1021/acsnano.8b07045

[117] B. Ding , S. Shao , C. Yu , B. Teng , M. Wang , Z. Cheng , K.‐L. Wong , P. Ma , J. Lin , Adv. Mater. 2018, 30, 1802479. 10.1002/adma.201802479 30387197

[118] C. Xu , J. Nam , H. Hong , Y. Xu , J. J. Moon , ACS Nano 2019, 13, 12148. 31556987 10.1021/acsnano.9b06691 PMC6832743

[119] G. Yang , L. Xu , J. Xu , R. Zhang , G. Song , Y. Chao , L. Feng , F. Han , Z. Dong , B. Li , Z. Liu , Nano Lett. 2018, 18, 2475. 29565139 10.1021/acs.nanolett.8b00040

[120] X. Wang , X. Zhong , Z. Liu , L. Cheng , Nano Today 2020, 35, 100946.

[121] Z. Li , L. Rong , Biomater. Sci. 2020, 8, 6272. 33016289 10.1039/d0bm01168a

[122] C. Ong , B. G. Cha , J. Kim , ACS Appl. Bio Mater. 2019, 2, 3630. 10.1021/acsabm.9b00483 35030750

[123] A. Seth , H. G. Derami , P. Gupta , Z. Wang , P. Rathi , R. Gupta , T. Cao , J. J. Morrissey , S. Singamaneni , ACS Appl. Mater. Interfaces 2020, 12, 42499. 32838525 10.1021/acsami.0c10781 PMC7942218

[124] M. Qian , L. Chen , Y. Du , H. Jiang , T. Huo , Y. Yang , W. Guo , Y. Wang , R. Huang , Nano Lett. 2019, 19, 8409. 31682447 10.1021/acs.nanolett.9b02448

[125] Y. Zhang , H. Chen , H. Wang , T. Wang , H. Pan , W. Ji , J. Chang , Chem. Eng. J. 2020, 380, 122472.

[126] W. Zhang , C. Zhang , X.‐Y. Wang , L. Li , Q.‐Q. Chen , W.‐W. Liu , Y. Cao , H.‐T. Ran , ACS Appl. Mater. Interfaces 2020, 12, 48420. 33073973 10.1021/acsami.0c16526

[127] Y. Cheng , Q. Chen , Z. Guo , M. Li , X. Yang , G. Wan , H. Chen , Q. Zhang , Y. Wang , ACS Nano 2020, 14, 15161. 33143424 10.1021/acsnano.0c05392

[128] F. Li , Y. Chen , S. Liu , X. Pan , Y. Liu , H. Zhao , X. Yin , C. Yu , W. Kong , Y. Zhang , Int. J. Nanomed. 2019, 14, 9917. 10.2147/IJN.S226466 PMC6927268 31908449

[129] M. N. Yukuyama , G. L. B. de Araujo , A. de Souza , R. Löbenberg , E. J. Barbosa , M. A. B. Henostroza , N. P. da Rocha , I. F. de Oliveira , B. R. Folchini , C. M. Peroni , J. F. Masiero , N. A. Bou‐Chacra , Int. J. Pharm. 2020, 587, 119697. 32750440 10.1016/j.ijpharm.2020.119697

[130] J. G. Croissant , Y. Fatieiev , N. M. Khashab , Adv. Mater. 2017, 29, 1604634. 10.1002/adma.201604634 28084658

[131] R. R. Castillo , M. Vallet‐Regí , Int. J. Mol. Sci. 2019, 20, 929. 10.3390/ijms20040929 PMC6413128 30791663

[132] T. I. Janjua , Y. Cao , C. Yu , A. Popat , Nat. Rev. Mater. 2021, 6, 1072. 34642607 10.1038/s41578-021-00385-x PMC8496429

[133] A. C. Eifler , C. S. Thaxton , in Nanoparticle Therapeutics: FDA Approval, Clinical Trials, Regulatory Pathways and Case Study (Ed: S. J. Hurst ), Humana Press, Totowa, NJ 2011, pp. 325–338. 10.1007/978-1-61779-052-2_21 21424459

[134] J. Liu , R. Zhang , Z. P. Xu , Small 2019, 15, 1900262.

[135] C. Wang , Y. Ye , Q. Hu , A. Bellotti , Z. Gu , Adv. Mater. 2017, 29, 1606036. 10.1002/adma.201606036 28556553

